# mitoXplorer, a visual data mining platform to systematically analyze and visualize mitochondrial expression dynamics and mutations

**DOI:** 10.1093/nar/gkz1128

**Published:** 2019-12-04

**Authors:** Annie Yim, Prasanna Koti, Adrien Bonnard, Fabio Marchiano, Milena Dürrbaum, Cecilia Garcia-Perez, Jose Villaveces, Salma Gamal, Giovanni Cardone, Fabiana Perocchi, Zuzana Storchova, Bianca H Habermann

**Affiliations:** 1 Max Planck Institute of Biochemistry, Am Klopferspitz 18, 82152 Martinsried, Germany; 2 Aix-Marseille University, INSERM, TAGC U1090, 13009 Marseille, France; 3 Aix-Marseille University, CNRS, IBDM UMR 7288, 13009 Marseille, France; 4 Functional Genomics of Mitochondrial Signaling, Gene Center, Ludwig Maximilian University (LMU), Munich, Germany; 5 Department of Molecular Genetics, TU Kaiserslautern, Paul Ehrlich Strasse 24, 67663 Kaiserslautern, Germany

## Abstract

Mitochondria participate in metabolism and signaling. They adapt to the requirements of various cell types. Publicly available expression data permit to study expression dynamics of genes with mitochondrial function (mito-genes) in various cell types, conditions and organisms. Yet, we lack an easy way of extracting these data for mito-genes. Here, we introduce the visual data mining platform mitoXplorer, which integrates expression and mutation data of mito-genes with a manually curated mitochondrial interactome containing ∼1200 genes grouped in 38 mitochondrial processes. User-friendly analysis and visualization tools allow to mine mitochondrial expression dynamics and mutations across various datasets from four model species including human. To test the predictive power of mitoXplorer, we quantify mito-gene expression dynamics in trisomy 21 cells, as mitochondrial defects are frequent in trisomy 21. We uncover remarkable differences in the regulation of the mitochondrial transcriptome and proteome in one of the trisomy 21 cell lines, caused by dysregulation of the mitochondrial ribosome and resulting in severe defects in oxidative phosphorylation. With the newly developed Fiji plugin mitoMorph, we identify mild changes in mitochondrial morphology in trisomy 21. Taken together, mitoXplorer (http://mitoxplorer.ibdm.univ-mrs.fr) is a user-friendly, web-based and freely accessible software, aiding experimental scientists to quantify mitochondrial expression dynamics.

## INTRODUCTION

Enormous amounts of transcriptomic data are publicly available for exploration. This richness of data gives us the unique opportunity to explore the behavior of individual genes or groups of genes within a vast variety of different cell types, developmental or disease conditions or in different species. By integrating these data in a sophisticated way, we may be capable to discover new dependencies between genes or processes.

Specific databases are available for mining and exploring disease-associated data, such as The Cancer Genome Atlas (TCGA, https://portal.gdc.cancer.gov/) (1), or the International Cancer Consortium Data Portal (ICGC, https://dcc.icgc.org/) (2). Especially cancer data portals allow users to perform deeper exploration of expression changes of individual genes or gene groups in different tumor types ((1–3); for a review on available cancer data portals, see (4)). Expression Atlas (https://www.ebi.ac.uk/gxa/home) on the other hand provides pre-processed data from a large variety of different studies in numerous species (5). Indeed, the majority of transcriptomic datasets are not related to cancer and are stored in public repositories such as Gene Expression Omnibus (GEO, https://www.ncbi.nlm.nih.gov/geo/) (6), DDBJ Omics Archive (https://www.ddbj.nig.ac.jp/gea/index-e.html) (7), or ArrayExpress (https://www.ebi.ac.uk/arrayexpress/) (8). Currently, it is not straightforward to integrate data from these repositories without at least basic programming knowledge.

Next to extracting reliable information from -omics datasets, it is equally important to support interactive data visualization. This is a key element for a user-guided exploration and interpretation of complex data, facilitating the generation of biologically relevant hypotheses—a process referred to as visual data mining (VDM, reviewed e.g. in (9)). Therefore, essentially all online data portals provide graphical tools for data exploration.

What is fundamentally lacking is a user-centric, web-based and interactive platform for data integration of a set of selected genes or proteins sharing the same cellular function(s). The benefits of such a tool are evident: first, it would give us the possibility to explore the expression dynamics and the presence of mutations in this set of selected genes across many different conditions, tissues and species. Second, by integrating data using enrichment techniques, for instance with epigenetic data or by network analysis using the cellular interactome(s), it would allow us to identify the mechanisms that regulate the expression dynamics of the selected gene set.

One interesting set of genes are mitochondria-associated genes (mito-genes): in other words all genes, whose encoded proteins localize to mitochondria and fulfill their cellular function within this organelle. Mito-genes are well-suited for such a systematic analysis, because we have a relatively complete knowledge of their identity and can categorize them according to their mitochondrial functions (10). This *a priori* knowledge can help us in mining and exploring the expression dynamics of mito-genes and functions in various conditions and species.

Mitochondria are essential organelles in eukaryotic cells that are required for producing cellular energy in form of ATP and for numerous other metabolic and signaling functions (10). Attributable to their central cellular role, mitochondrial dysfunctions were found to be associated with a number of human diseases such as obesity, diabetes, neurodegenerative diseases and cancer (11–15). However, mitochondria are not uniform organelles. Their structural and metabolic diversity, both of which influence each other, has been well described in literature (16–20). This mitochondrial heterogeneity in different tissues is reflected in their molecular composition (21). The total number of proteins that contribute to mitochondrial functions and localize to mitochondria is currently not precisely known and might differ between tissues and species (22,23). Yet, based on proteomic data from several organisms, it is likely that mitochondria contain >1000 proteins (23–30). Mitochondria have their own genome, whose size in animals is between 11 and 28 kb (31). Most metazoan mitochondria encode 13 essential proteins of the respiratory chain required for oxidative phosphorylation (OXPHOS), all rRNAs of the small and large mitochondrial ribosomal subunits, as well as most mitochondrial tRNAs (32). All other proteins found in mitochondria (mito-proteins) are encoded by genes in the nucleus; the protein products of these nuclear-encoded mitochondrial genes (NEMGs) are transported to and imported into mitochondria.

Based on data from mitochondrial proteomic studies or genome-scale prediction of mito-proteins, several electronic repositories of the mitochondrial interactome have been created (24,33–36), though they often lack a proper functional assignments of mito-proteins. Moreover, proteomic studies describing the mitochondrial proteome can suffer from a high false-positive rate (23), whereas computational prediction or machine learning in most cases lack experimental confirmation (37). As a consequence, none of the published mitochondrial interactomes available to date can be taken without further manual curation. Moreover, these lists are not integrated with any available data analysis tool to explore mitochondrial expression dynamics under varying conditions or in different tissues or species.

In this study, we present mitoXplorer, a web-based, highly interactive visual data mining (VDM) platform designed to specifically mine the dynamics of a manually curated gene set with mitochondrial functions in transcriptome, proteome, as well as mutation-based data. To achieve this, mitoXplorer integrates -omics data with our hand-curated mitochondrial interactomes for currently four different model species. With mitoXplorer, we can explore the expression dynamics, as well as mutations of mito-genes and their associated mitochondrial processes (mito-processes) across a large variety of different -omics datasets without the need of programming knowledge. MitoXplorer provides users with dynamic and interactive figures, which instantly display information on mitochondrial gene functions and protein-protein interactions. Users can analyze publicly available data stored in our mitoXlorer database or upload their own data for integration with our hand-curated mitochondrial interactome. In order to demonstrate the analytical and predictive power of mitoXplorer and to experimentally verify mitoXplorer predictions, we generated transcriptome and proteome data from aneuploid cell lines, carrying trisomy 21 (T21), the most common chromosome abnormality in humans which is also known to cause substantial mitochondrial dysfunctions (38). We used mitoXplorer to analyze and integrate our data with publicly available trisomy 21 data. MitoXplorer enabled us to predict respiratory failure in one of our T21 cell lines, which we experimentally confirmed, thus demonstrating the predictive power of mitoXplorer.

## MATERIALS AND METHODS

### Implementation of mitoXplorer

#### Web interface of mitoXplorer (front-end)

The web interface of mitoXplorer at the *front-end* allows users to access, interact and visualize data from its database, including the interactome and expression/mutation data. The interactive elements and visualizations on mitoXplorer are all built with Javascript, a dynamic programming language that enables interactivity on webpages by manipulating elements through DOM (Document Object Model). DOM is a representation of document, such as HTML, in a tree structure, with each element as a node or an object. Through Javascript and its libraries, visualizations in mitoXplorer can react to users’ action and dynamically change the properties (size, color, coordinates) of web elements and display interactivity. All the visualization components in mitoXplorer described below are modular by design and can be deployed individually or incorporated into web platforms easily.

##### Mitochondrial Interactome (D3—data binding and selection)

The visualization of the interactome is created with the implementation of a Javascript library, D3 (d3.js) (39). D3 (data-driven documents) is capable of binding data, usually in JSON (Javascript-oriented notation) format, to the elements of the DOM so that their properties are entirely based on given data. In the Interactome View, D3 creates an SVG (Scalable Vector Graphic) element for each gene within the DOM in the form of a bubble, with sizes and colors dependent on the associated log2 fold change (log2FC) values. The coordinates of bubbles are also calculated according to the data (e.g. the largest one being at the center) so that the layout of the whole interactome is visually appealing. Upon hovering over any bubble (gene), D3 selects the element and passes additional data bound to that element to the corresponding web element (sidebar) for display.

##### Comparative plot (D3—transition and sorting)

The comparative plot combines three interdependent visualizations (scatterplot, bar chart and heatmap) built upon D3. Apart from data-binding and selection, these visualizations exploit the functionality of D3 for transition and sorting through its API. In the scatterplot, genes are displayed as nodes, whose colors and positions again depend on the data (log2FC). When another mito-process is selected at the bar chart, D3 updates the data bound to the node and the properties of the nodes are changed. The transition (changes in color and position) is smooth and gives users the impression that the visualization is truly dynamic and interactive. D3 can manipulate not only the elements, but also the data bound to the elements. Upon clicking the dataset or gene names on the heatmap, the data can be sorted accordingly and an index is assigned to each element (tile on the heatmap) to indicate its position.

##### Hierarchical clustering (mpld3—visualization in Python implemented in D3)

The heatmap displaying the results of hierarchical clustering is built with mpld3, a Python library that exports graphics made with Python's Matplotlib-based libraries to JSON objects that can be displayed on web browsers. Mpld3 benefits from D3’s data-binding property and allows users to create a plugin that interacts with the data on the visualization. The advantage of using mpld3 is that analyses and visualizations made in Python can be directly translated to JSON and deployed in Javascript on webpages without re-programming. In the case of hierarchical clustering, since libraries for both clustering analysis and visualization of results in a heatmap with a dendrogram are available in Python (described below), it is exported to JSON with mpld3 and a Javascript tooltip plugin that allows users to select data or display information with D3.

##### Principal component analysis (three.js—3D visualization)

The visualization of the result of Principal Component Analysis (PCA) is 3-dimensional, with each dimension representing one of the first three principal components (PCs). This is achieved through the implementation of three.js, a Javascript library that enables animated 3D graphics to be created and displayed in a web browser. It starts with building a ‘scene’, or a canvas, on which 3D objects will be created. Then a ‘camera’ is set up that controls the view of objects on the scene from the users’ perspective, such as the field of view (width, height, depth) and its ratio; and a ‘renderer’ that renders the scene at short time intervals so objects are displayed as animated objects (either they are animated by themselves or moved around on the scene by users). Objects of different texture, geometry and color can now be added to and rendered on the scene. Finally, the scene with objects is attached to the DOM of a webpage to become visible. In the PCA visualization, each dataset is represented and rendered as a small sphere, with coordinates (*x*, *y*, *z*) depending on the values of its first three PCs, and colors on the grouping of that dataset. When users drag around on the canvas or zoom in or out, all objects are re-rendered in such a way that the scene appears to be a 3-dimensional space.

#### MitoXplorer database (*back-end*)

A MySQL database hosted at the *back-end* of mitoXplorer contains the interactomes of mito-genes, including the mito-process, gene ontology and the interactions between gene products; and the expression and mutation data from public databases. Each entry of the expression and mutation data has a foreign link to the interactome and file directory (dataset table). This ensures that the expression and mutation data will be updated together with the interactome, or when a dataset is updated or deleted. Users can upload their own differential expression and/or mutation data, which will be processed and integrated with the interactome by extracting mito-genes, and stored in the mitoXplorer database for up to 7 days.

#### Data analysis and communication between *front*- and *back-end*

A Python application serves as a bridge between the *front*- and *back-end* of mitoXplorer. Upon the users’ request to access the database or perform analysis at the web interface, an AJAX-asynchronous call directed to the Python application is made, so the request can be performed in the background and the webpage is updated without reloading. The Python application then processes the request by connecting to the MySQL database and analyzes the data retrieved from it. The application also handles the user uploads (e.g. data cleaning) before saving it to the MySQL database. The main libraries used by the Python application for analysis include: (i) Scikit-learn: a machine learning library that provides tools for PCA, to perform dimensionality reduction on the expression of all mito-genes and of each mito-process. The first three principal components are extracted for each dataset. (ii) SciPy: a mathematical library that provides modules for hierarchical clustering, to calculate 2D distance matrices between genes and between datasets based on expression values, for each mito-process. (iii) Seaborn: a statistical visualization library built on top of SciPy to create heatmaps from the results. All the results are produced in JSON format, which are then sent via the HTTP protocol back to the *front-end* and visualized with Javascript.

The usage of mitoXplorer does not require installation or programming knowledge. Documentation and tutorials are available online and on GitLab (https://gitlab.com/habermannlab/mitox). MitoXplorer is also available for download and installation on a local server, if users wish to build their own gene list and apply the interactive features and database of mitoXplorer, which stores the available expression and mutation data for all genes. Setup instructions are also available on the mitoXplorer GitLab repository, as is a docker version of mitoxplorer (https://gitlab.com/habermannlab/mitox, branch docker-version).

#### Processing of public transcriptomic and proteomic data

Proteomic and transcriptomic data from Kühl (40), as well as Liu (41), Letourneau (42), Sullivan (43) and Spletter (44) were uploaded as provided by the authors.

Public NGS datasets downloaded from GEO which did not already contain differential expression data in the form of log2FC and *P*-value were analyzed according to available pre-analyzed data: datasets with available raw read counts were analyzed using DESeq2 (version 3.9, (45)). These included data from Chowdhury (46) and Garipler (47). Datasets for which only the normalized read counts were available, the log_2_FC was calculated for each sample, using the corresponding wild-type samples as control (or the mean of normal samples if there were no paired samples) following best-practice guidelines. We applied this to data from TCGA (1), downloaded from the NCI GDC Data Portal (https://portal.gdc.cancer.gov/)), from Fleischer (48) and from Huang (49). Finally, microarray time-course data of yeast meisosis (GEO accession: GSE75257) were analyzed with GEO2R (50).

Metadata of the datasets (e.g. cell types, analysis pipeline and genome version used for mapping) were also downloaded and stored in the mitoXplorer database. The links to the experiments for each dataset are available at the DATABASE summary page of mitoXplorer.

#### Transcriptomics and proteomics of aneuploid cell lines

The proteome analysis of the trisomic cell lines was previously described (51,52).

The raw reads from RNA-sequencing were processed to remove low quality reads and adapter sequences (using TrimGalore v0.4.5 (https://www.bioinformatics.babraham.ac.uk/projects/trim_galore/), which uses Cutadapt (53)) and FastQC (Andrew, S. (2010) FastQC: a quality control tool for high throughput sequence data. http://www.bioinformatics.babraham.ac.uk/projects/fastqc)), and aligned to the human reference genome (version hg19) with TopHat2 (v2.0.11) (54). Cuffdiff from the Cufflinks package (v2.2.1) (55) was used with standard parameters to calculate the expression difference between two samples (aneuploid versus diploid) of multiple replicates and test the statistical significance. Transcriptome and proteome information are available in public repositories: NGS data have been deposited in NCBI’s Gene Expression Omnibus and are accessible through GEO series accession number GSE102855 and GSE131249.

#### Cell culture and treatment

The human cell line RPE-1 hTERT (referred to as RPE) was a kind gift by Stephen Taylor (University of Manchester, UK). Human HCT116 cells (referred to as HCT) were obtained from ATCC (No. CCL-247). Trisomic cell lines were generated by microcell-mediated chromosome transfer as described previously (51). The A9 donor mouse cell lines were purchased from the Health Science Research Resources Bank (HSRRB), Osaka 590-0535, Japan. All cell lines were maintained at 37°C with 5% CO_2_ atmosphere in Dulbecco′s modified Eagle's medium (DMEM) containing 10% fetal bovine serum (FBS), 100 U penicillin and 100 U streptomycin.

#### MitoTracker staining and imaging

Mitochondria were stained in 96-well plates. The cells were incubated for 30 min at 37°C with 100 nM MitoTracker deep Red FM (M22426, Invitrogen®) dye prior to fixation. Cells were fixed with 3% PFA in DMEM for 5 min at room temperature. After washing twice with 1xPBST, plates were stored with 1× PBS containing 0.01% sodium azide. Plates were stored at 4°C in the dark. Imaging was carried out on an inverted Zeiss Observer.Z1 microscope with a spinning disc and 473, 561 and 660 nm argon laser lines. Imaging devices were controlled, and images were captured, stored and processed with the SlideBook Software in Fiji (56). The images were captured automatically on multiple focal planes (step size: 700 nm) with a 40× magnification air objective.

#### Metabolic profiling of wild-type and T21 cell lines

RPE and HCT cells and their T21 derivatives were seeded at 25 000 or 36 000 cells/well respectively, on XF96 cell plates (Seahorse Bioscience, Agilent Technologies), 30 h before being assayed. Optimization of reagents as well as CCCP and digitonin titrations were performed as described by the manufacturer's protocols (Seahorse Bioscience). The experiments were performed using the mitochondrial and glycolytic stress test assay protocol as suggested by the manufacturer (Seahorse Bioscience, Agilent Technologies). By employing the Seahorse Bioscience XF Extracellular Flux Analyzer, the rate of cellular oxidative phosphorylation (oxygen consumption rate (OCR)) and glycolysis (cellular proton production rate (PPR)) were measured simultaneously.

For OCR measurement, DMEM media was supplemented with 25 mM glucose, 1 mM pyruvate and 2 mM glutamine. Basal rate was recorded and additions for the mito stress test were as follows: 1.5 μM oligomycin, CCCP, 2 μM rotenone + 4 μM antimycin A. For PPR measurement, DMEM media was supplemented with 2 mM glutamine. Basal rate was recorded and additions for the glycolysis stress test were as follows: 10 mM glucose, 1.5 μM oligomycin and 100 mM 2-deoxyglucose.

For intact cells, the CCCP concentrations were 7 and 1.5 μM for RPE1 and HCT116 cells, respectively. The assays of intact cells were performed in 96-well plates with at least 10 replicates per cell line. For the permeabilized RPE1 cell lines, the CCCP and digitonin concentrations were 10 and 40 μM, respectively. For OCR measurement, Mannitol–sucrose buffer (MAS) was prepared according to Seahorse Biosciences. For permeabilization, digitionin was added to MAS buffer together with the respective respiratory substrates: 10 mM pyruvate/2 mM malate, 10 mM succinate/2 μM rotenone or 0.5 mM TMPD/2 mM ascorbate/2 μM antimycin A. Basal respiration was recorded, as were additions of 4 mM ADP, 1.5 μM oligomycin, CCCP and 2 μM rotenone ± 4 μM antimycin A or 20 mM Na-azide. The assays in permeabilized cells were performed in poly-d-lysine-coated 96-well plates with at least five replicates per cell line.

Normalization was performed with the CyQuant cell proliferation assay kit (Life Technologies) in the same plate used for the assay of intact cells; and in a parallel plate for the permeabilized cells. Data analysis was done according to (57).

#### The mitoMorph plugin for morphological characterization of mitochondria by image analysis

Classification and measurement of mitochondria were performed using the software ImageJ (58), complemented with all the default plugins provided by Fiji (56) and with the additional plugin FeatureJ. A set of functions were developed to assist the user in the preparation and analysis of the data, either in interactive or batch processing mode.

Using this toolset, after all the cells of interest were manually outlined in each image, the mitochondria were segmented and characterized. For each processing step, the algorithms used are reported as described in ImageJ, and their parameters are specified in physical units.

The images were pre-processed by first suppressing the background signal (rolling ball background subtraction, kernel radius: 2.5 μm) and then enhancing the mitochondria signal (Laplacian of Gaussian, smoothing scale: 1 μm, followed by contrast limited adaptive histogram equalization, CLAHE, kernel size: 2.5 μm). Mitochondria candidates were obtained by segmentation, using Yen thresholding algorithm (59), and subjected to classification based on a set of determined features.

Objects that were too small were excluded from the analysis, and the remaining ones were assigned to one of four categories: filamentous networked (filaments), puncta, rods and swollen (60). Objects that were quasi-round, compact in intensity, and larger than the puncta were classified as swollen. All objects with an intermediate phenotype between fragmented puncta and network of filaments were classified as rods.

Classification was performed by sequentially verifying different selection criteria, one set for each class, based on the following measured features: area (A), aspect ratio (AR), circularity (C), solidity (S), minimum Feret diameter (here indicated as minimum linear extension, MLE) and longest shortest-path (here indicated as extension, E). While all the other measures are directly derived from the segmentation, the extension is measured as the longest shortest-path between any two end points in the skeleton derived from the segmentation. The selection criteria are evaluated sequentially as reported in Supplementary Table S1.

We would like to note that analysis of mitochondrial morphology on projected images is limited, as mitochondrial structures might not be resolved properly.

#### Image analysis using mitoMorph and data processing

Image processing and analysis was done in Fiji. Image stacks were *Z*-projected, cells were manually selected and the resulting images were saved for further batch processing using mitoMorph. Resulting network statistics of mitochondrial features for each individual cell were used for further processing (Supplementary Table S1). All statistical processing and data visualization of mitoMorph results was done using RStudio (v1.1.423, R-version: 3.6.1). Data were averaged over both clones of the two T21 cell lines.

## RESULTS

**Figure 1.**
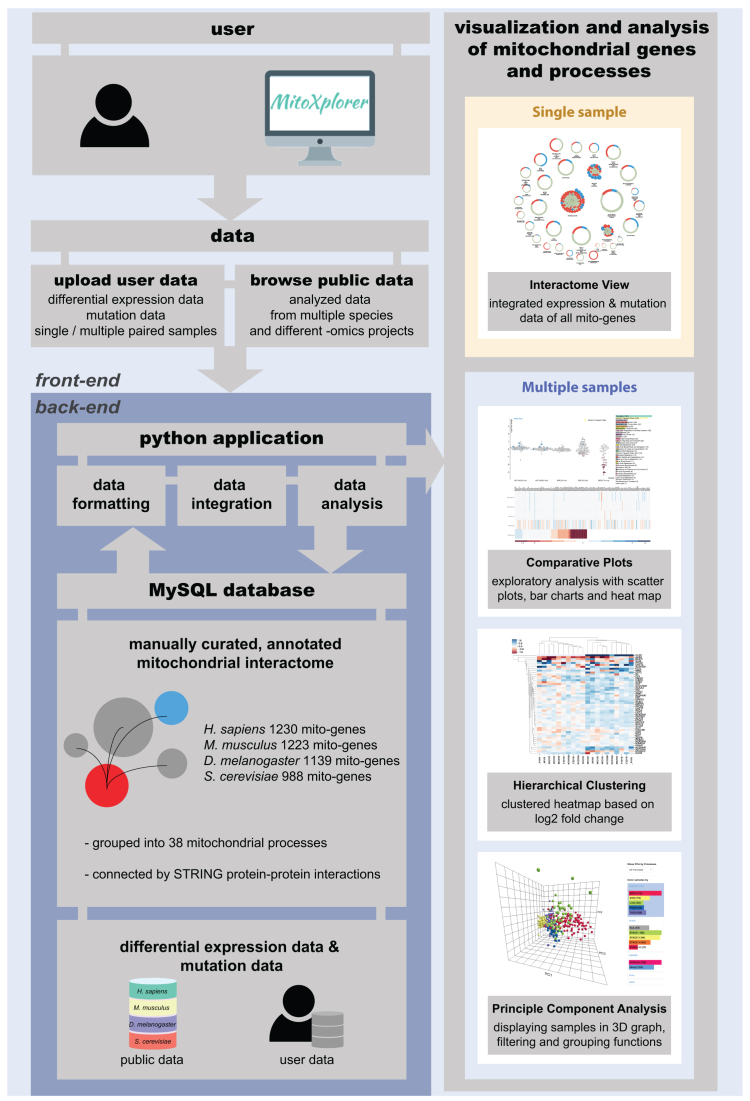
Setup of the mitoXplorer web-based visual data mining platform. A manually curated, annotated mitochondrial interactome represents the central part of the mitoXplorer software, for which we have assembled 1229 mito-genes in human, 1222 mito-genes in mouse, 1139 mito-genes in fruit fly and 988 mito-genes in budding yeast in 38 mitochondrial processes (mito-processes). We have connected gene products using protein-protein interactions from STRING (69). Publicly available expression and mutation data from repositories such as TCGA or GEO are provided for data integration, analysis and visualization and are stored together with species interactomes in a MySQL database. Users can provide their own data, which are temporarily stored and only accessible to the user. A set of Python-based scripts at the *back-end* of the platform handle data formatting, integration and analysis (Supplementary Figure S1). The user interacts with mitoXplorer via several visual interfaces to analyze, integrate and visualize his private, as well as public data. Four interactive visualization interfaces are offered: (i) the Interactome View allows at-a-glance visualization of the entire mitochondrial interactome of a single dataset (see Figure 2); (ii) comparative plots, consisting of a scatterplot and a sortable heatmap allows comparison of up to six datasets, whereby a single mito-process is analyzed at a time (see Figure 3); (iii) hierarchical clustering allows comparison of a large number of datasets which are clustered according to their expression values. Hierarchical clustering plots are zoom-able and interactive (see Figure 4); (iv) principal component analysis displays PCA-analyzed datasets in 3D, providing filtering and grouping functions. There is in principle no limit to the number of datasets that can be analyzed using PCA (see Figure 5).

The outline of the mitoXplorer web-platform is illustrated in Figure 1: at the *back-end*, manually curated mitochondrial interactomes from human, mouse, *Drosophila* and budding yeast, as well as expression and mutation data from these four species are stored in a MySQL database (details on the implementation of the *back-end* are available in Materials and Methods, as well as Supplementary Figure S1).

The user interacts with the mitoXplorer web-platform via the *front-end*, which offers different visualization and analysis methods. Users can either browse stored public data or upload their own data.

### The mitochondrial interactomes

The main component of mitoXplorer is the mitochondrial interactome. Its accurate annotation and completeness are essential for performing a meaningful mitoXplorer-based analysis. To establish mitochondrial interactomes, we have assembled and manually curated lists of genes with annotated mitochondrial processes (mito-processes). Currently, the interactomes of four organisms are available on mitoXplorer: *Homo sapiens* (human), *Mus musculus* (mouse), *Drosophila melanogaster* (fruit fly) and *Saccharomyces cerevisiae* (budding yeast). We started from published mitochondrial proteomic data (27,61) for the selected species and manually cleaned the data, as well as supplemented missing mito-genes in the following way: we removed obvious false-positives from the datasets; these included mainly proteins for which there is compelling evidence in literature that they are not localized to mitochondria. Next, we supplemented likely missing genes from the proteomic data using information from Mitocarta (24), KEGG (62) information of genes associated with mitochondrial pathways, as well as orthologs across species from the four proteomic datasets. To establish whether a protein in question is primarily localized to mitochondria or not, we relied on several sources: (i) evidence from the literature on a specific gene; (ii) information from the respective gene entry at NCBI (63), Flybase (64) or the *Saccharomyces* genome database (SGD) (65); (iii) information from the GeneCards database (66); (iv) information from UniProt (67). We supplemented the lists with all non-coding genes present in the mitochondrial genomes, namely all mitochondrial rRNAs, as well as tRNAs. After manual curation, we obtained 1229 human, 1222 mouse, 1139 *Drosophila* and 988 budding yeast mito-genes. We grouped the genes in mito-processes using controlled vocabulary. In addition to purely mitochondrial processes, we added cytosolic processes coupled to mitochondrial functions, including Glycolysis, the Pentose phosphate pathway, Apoptosis or the regulation of transcription of nuclear-encoded mitochondrial genes (Transcription (nuclear)). This resulted in a total of 38 mito-processes (Table 1). We selected the correct mito-process for each gene primarily using information from the same sources mentioned before (NCBI gene entry, Flybase, SGD, GeneCards, UniProt, KEGG). According to our current annotation, one gene is part of only a single mito-process. We acknowledge that this annotation strategy has limitations, as a mito-gene can be part of more than one mito-process: as an example, in KEGG, the human gene GPI is part of the pathways Glycolysis, as well as Pentose phosphate pathway. In GeneCards, GPI is primarily associated with Glycolysis, thus we assigned it to the mito-process Glycolysis. For other genes, the existing annotations were less clear and we had to decide on the primary process a gene should belong to. For some mito-processes, we solved this by introducing the ontologically higher ranked term: for instance, mito-genes involved in fatty acid (FA) metabolic pathways can be associated with either the biosynthesis or degradation of FAs, or both: genes implicated in both processes were allocated to the term that would rank higher, namely Fatty acid metabolism. Genes involved in the transport of molecules across the mitochondrial membrane were divided into three groups: those involved in import & sorting of all mito-proteins that are encoded in the nuclear genome and translated in the cytosol into mitochondria; those that are part of the mitochondrial carrier family (68); and finally those transmembrane proteins that are involved in mitochondrial transmembrane transport and cannot be associated with either of the other two groups. Finally, to complete the mitochondrial interactome and reveal potential interactions between mito-processes, we added protein-protein interaction information from STRING (69) for all mito-genes.

**Table 1.** Mito-processes and number of associated mito-genes in human, mouse, *Drosophila* and budding yeast

Mito-process	Human	Mouse	*Drosophila*	Budding yeast
**Amino acid metabolism**	81	79	67	44
**Apoptosis**	56	55	43	6
**Bile acid synthesis**	2	2	7	0
**Calcium signaling & transport**	23	23	12	4
**Cardiolipin biosynthesis**	6	6	5	5
**Fatty acid biosynthesis & elongation**	22	22	15	13
**Fatty acid degradation & beta-oxidation**	30	31	26	9
**Fatty acid metabolism**	15	13	20	8
**Fe-S cluster biosynthesis**	25	26	19	23
**Folate & pterin metabolism**	13	13	9	13
**Fructose metabolism**	7	7	3	14
**Glycolysis**	38	37	35	33
**Heme biosynthesis**	9	9	9	5
**Import & sorting**	51	51	61	55
**Lipoic acid metabolism**	3	3	4	3
**Metabolism of lipids & lipoproteins**	34	36	17	14
**Metabolism of vitamins & co-factors**	17	18	19	9
**Mitochondrial carrier**	46	45	46	23
**Mitochondrial dynamics**	61	59	48	39
**Mitochondrial signaling**	18	18	10	11
**Mitophagy**	21	21	13	11
**Nitrogen metabolism**	9	9	16	7
**Nucleotide metabolism**	15	15	12	23
**Oxidative phosphorylation**	167	164	173	115
**Oxidative phosphorylation (MT)**	13	13	13	9
**Pentose phosphate pathway**	7	7	6	14
**Protein stability & degradation**	27	27	20	25
**Pyruvate metabolism**	26	25	24	12
**Replication & transcription**	51	52	32	50
**ROS defense**	34	34	30	24
**Transcription (nuclear)**	24	24	25	6
**Translation**	185	184	192	210
**Translation (MT)**	24	24	24	37
**Transmembrane transport**	20	20	21	24
**Tricarboxylic acid cycle**	21	22	29	26
**Ubiquinone biosynthesis**	9	9	9	12
**Unknown**	12	12	20	46
**UPRmt**	7	7	4	6

Since we cannot guarantee that our current annotation is either complete or exempt from mis-annotations, and with the goal of nucleating a community-based effort to further complete and improve the annotation of the mitoXplorer mito-interactomes, we provide a ‘FEEDBACK’ page. Users can submit comments and suggestions on genes and their annotations using this page, suggest new genes and new mito-processes or provide any other feedback.

Mito-genes of human, mouse, *Drosophila* and budding yeast annotated with mito-processes are available in Supplementary Table S2A–D. The mito-interactomes can also be downloaded from the INTERACTOME page of mitoXplorer. These manually curated and annotated interactomes enable a meaningful analysis and visualization of mitochondrial expression dynamics of mito-genes and mito-processes by comparing differential expression of two or more conditions in mitoXplorer.

### The mitoXplorer expression and mutation database

To foster the analysis of mitochondrial expression dynamics and mutations, mitoXplorer hosts expression and mutation data from public repositories in a MySQL database.

Expression data encompass analyzed data of differentially expressed genes from RNA-seq studies and are available in the form of log_2_ fold change (log2FC) and *P*-value. One differential dataset thus includes two experimental conditions with all replicates. Mutation data include analyzed data of identified SNPs of one sample against a publicly available reference genome or transcriptome.

Pre-analyzed public data are taken as provided by the authors of the respective study: information on software and genome version used for read mapping, as well as software and settings used for differential expression analysis can generally be found at the GEO-link of the respective project listed on the DATABASE page. We have ensured that only high-quality data with replicates, as well as a properly described analysis strategy are available in the mitoXplorer database. If only raw read counts were available, we analyzed the data using state-of-the-art software (DESeq2 (45), for details see Methods). Finally, whenever only normalized read counts were available, which is typical for large population-based studies, we calculated log2FC according to (70). It should be noted that due to the heterogeneity of the available formats of the provided data, the algorithms and their settings, as well as the genome version used for read mapping might differ for available projects in mitoXplorer.

The largest public resource imported into mitoXplorer covers publicly available expression data of human cancers from The Cancer Genome Atlas (TCGA) (1). We have included all paired samples. This resulted in a total of 523 differential datasets from six different cancer types: kidney cancer (KIRK), breast cancer (BRCA), liver cancer (LIHC), thyroid cancer (THCA), lung cancer (LUAD) and prostate cancer (PRAD). Changes in mitochondrial metabolism have been described in many cancer types (for a review, see (71)). As mitoXplorer is thus far the only resource that allows a focused analysis of mito-genes across different cancer types or patient groups, this resource should be especially useful to shed light on the expression dynamics of mito-genes in cancer and to classify the mitochondrial metabolic profiles of tumor types and sub-types. Users can moreover integrate proprietary data with differential expression data from different tumor types and subtypes.

We also uploaded expression data from cultivated fibroblasts of healthy human donors ranging from 1 to 94 years of age (48) (GEO accession: GSE113957). Since decline in mitochondrial quality and activity are well-known contributors to age-related conditions and diseases (72), this dataset should help uncover the contribution of altered mito-gene expression dynamics to the ageing process.

We made available several datasets from mouse knock-out studies: we uploaded differential transcriptomic and proteomic data of five different mouse conditional heart knock-out strains of genes involved in mitochondrial replication, transcription and translation (40) (Lrpprc, Mterf4, Tfam, Polrmt, Twnk (Twinkle), (GEO accession: GSE96518)). These data are especially helpful in unraveling the transcriptional and post-transcriptional effects on mito-genes upon disruption of gene expression at different levels in mitochondria.

Furthermore, we added data from a mouse model of a known mitochondria-associated condition, the Barth syndrome. Barth syndrome patients develop severe cardiomyopathy (73). This syndrome is caused by mutations in or loss of the TAZ gene coding for the protein Tafazzin which is involved in cardiolipin biosynthesis (74). Failure of enzyme activity of Tafazzin leads to altered mitochondrial membrane composition, structure and metabolism (74,75). We provide differential expression data of Taz knock-out mouse embryonic fibroblasts (MEFs) compared to wild-type in normoxic and hypoxic conditions generated by Chowdhury *et al.* (46) (GSE accession: GSE119775); these data should help reveal the effect of Tafazzin loss of function during hypoxia.

To extend mitoXplorer to other model organisms, we added data from *D. melanogaster*, namely expression data from 185 wild-derived, inbred strains (males and females) from the *Drosophila* Genetics Reference Panel (DGRP2) (49): this set of lines stems from an out-crossed population in Raleigh, North Carolina. These wild-derived fly strains display a substantial quantitative genetic variation in gene expression. The availability of these data on mitoXplorer allows a focused analysis of mito-genes to elucidate whether mitochondrial expression dynamics is equally impacted in these strains.

Moreover, we have uploaded data from a recently published systematic study of flight muscle development in *D. melanogaster* (44) (GEO accession: GSE107247). This enables the analysis of mitochondrial expression dynamics during the development and differentiation of a tissue that is highly dependent on an efficient mitochondrial metabolism and especially ATP production for proper functioning.

Regarding budding yeast, we imported data from a time-course expression profiling experiment of meiosis of a synchronized cell culture (Hanlon SE, Lieb JD (unpublished), GEO accession: GSE75257), allowing users to mine the expression dynamics of mito-genes over 12h of sporulation. This project is the only microarray-based dataset we have uploaded on mitoXplorer.

Finally, we uploaded data from Protein Phosphates 2A (PP2A) yeast deletion strains; these strains show a diminished response of nuclear gene expression associated with mtDNA damage compared to wild-type (47) (GEO accession: GSE52242). This dataset should help shed light on the role of the conserved protein phosphatase PP2A in protecting cells from mtDNA damage.

To verify mitoXplorer predictions experimentally, we use data from human trisomy 21: we provide data from human trisomy 21 patients (GEO accession numbers: GSE55426; GSE79842; (42,43)), from trisomy 21 studies in mouse (GSE5542 (42), GSE79842 (43)), as well as differential datasets generated in the course of this study from human trisomic cell lines (11 datasets) which have been partially published elsewhere (51,52) (GEO accessions: GSE39768; GSE47830; GSE102855). These transcriptomic, as well as proteomic datasets should help understand the role of mitochondria and the mitochondrial metabolism in trisomy 21.

All available data can be viewed and accessed from the mitoXplorer DATABASE web-page.

### User-provided expression and/or mutation data

Researchers can upload and explore their own data in mitoXplorer, given that they originate from one of the species contained in the mitoXplorer platform. Data must be pre-analyzed. Differential expression data must contain the dataset ID (describing the experimental condition), the gene name and the log2FC. Optional values include the *P*-value, as well as the averaged read counts (or intensities) of the replicates of the compared conditions. Mutation data must contain the dataset ID, gene name, the chromosome, the position, as well as reference and alternative allele. Optional values include the effect, as well as the consequence of the mutation. Users have the option to either generate their own data according to the format described on our website. We recommend to follow the best-practice-guidelines available for analyzing transcriptomic or proteomic data prior to uploading data to mitoXplorer (see for instance (76,77) for differential expression analysis or (78) for variant calling). Alternatively, users may use the RNA-seq pipeline for differential expression analysis and mutation calling that we provide at https://gitlab.com/habermannlab/mitox_rnaseq_pipeline/.

The entire list of genes from a study should be uploaded to the platform for several reasons: first, a restriction to only differentially expressed or mutated genes will suppress links between proteins in the interactome; second, an integration of user data with publicly provided data is difficult with incomplete datasets; third, mitoXplorer will automatically select the mito-genes from the user data. Uploaded data will be checked for correct formatting and integrated with the interactome of the chosen species. User data are only visible to the owner and are stored in the mitoXplorer MySQL database for 7 days. Users can integrate their own data with available public data on mitoXplorer to perform various analyses and visualizations as described below (Figure 1).

### Analysis and visualization tools in mitoXplorer

The mitoXplorer web-platform provides a set of powerful, easy-to-read and highly interactive visualization tools to analyze and visualize public, as well as user-provided data by VDM (Figure 1): an **Interactome View** to analyze the overall expression and mutation dynamics of all mito-processes of a single dataset containing differentially expressed genes between two conditions and potential mutations in mito-genes; the **Comparative Plot**, consisting of an interactive scatterplot, as well as an interactive heatmap for comparing up to six datasets; the **Hierarchical Clustering**, as well as the **Principal Component Analysis** for comparative analysis of many datasets.

## INTERACTOME VIEW

**Figure 2.**
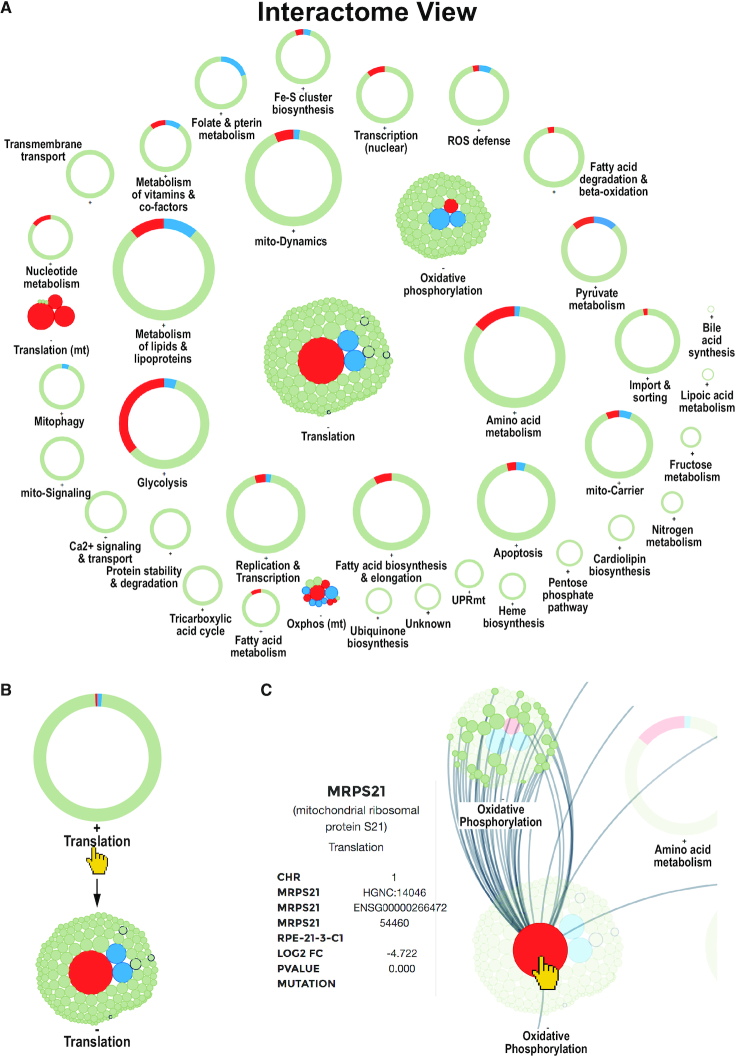
Interactome View of the mitoXplorer platform. (**A**) Overview of all mito-processes of one dataset. A process can either be shown as one circle with colored segments according to the number of dysregulated genes, or upon clicking on the process, by showing all individual genes being part of this process, see (**B**): by clicking on the process Translation or the adjacent ‘+’, the circle is replaced by individual bubbles representing genes of this process. Clicking on the process again, or on the adjacent ‘–’ will revert to the circular display. (**C**) Hovering over a gene bubble will display the name of the gene and associated information (gene name, description, chromosomal location, mitochondrial process, accession numbers, as well as log_2_ fold change, *P*-value and observed mutations), as well as all connections to mito-genes in other processes. Compared were the retinal epithelial cell line RPE1 (RPE) wild-type to RPE1 with Trisomy 21 (RPE_T21).

The Interactome View can be used to get an at-a-glance view of the overall expression dynamics of all mito-processes of a single dataset of differentially expressed mito-genes and potential mutations (Figure 2A). It allows users to identify the most prominently changed mito-processes or -genes in a dataset. The genes are grouped according to mito-processes and displayed in the process they are assigned to. The Interactome View is highly dynamic and can be adjusted by users to their needs.

When the Interactome View is launched, each mito-process is primarily shown as a grey circle with elements colored in grey, blue and/or red, indicating up- or down-regulated genes within the process, respectively (Figure 2A). Thus, mito-processes with the most up- or down-regulated genes can be quickly identified.

When clicking on a process name, its circle opens up to display all its member genes as bubbles. The size of the bubble relates to the strength of the differential regulation while the color indicates up- (blue) or down- (red) regulation of the gene (Figure 2B). If information about mutations is included in the dataset, this is indicated by a thicker, black border of the gene bubble.

Hovering over a gene will display the gene name, its function, its mito-process, the log2FC and the *P*-value of the differential expression analysis, as well as potential mutations in the *information panel* (Figure 2C). If a gene physically interacts with other mito-genes, hovering over it or over the process circle will in addition display these connections (Figure 2C). Thus, the user is immediately informed about the location and connectivity of the protein of interest within the mitochondrial interactome. Users can also search for specific genes using the ‘FIND A GENE’ box at the top of the page.

The Interactome View can be launched by clicking on the ‘eye’ symbol next to dataset names from the ANALYSIS page of mitoXplorer, after having chosen the organism, the project and the dataset. Alternatively, users can access single datasets from the DATABASE page of the platform, by clicking on the eye symbol of a listed dataset after having chosen a species, as well as a project. A new page will be opened for the Interactome View, which allows opening and comparing multiple datasets at the same time. This is especially useful for comparing the overall expression change of mito-processes of multiple datasets.

## COMPARATIVE PLOT

**Figure 3.**
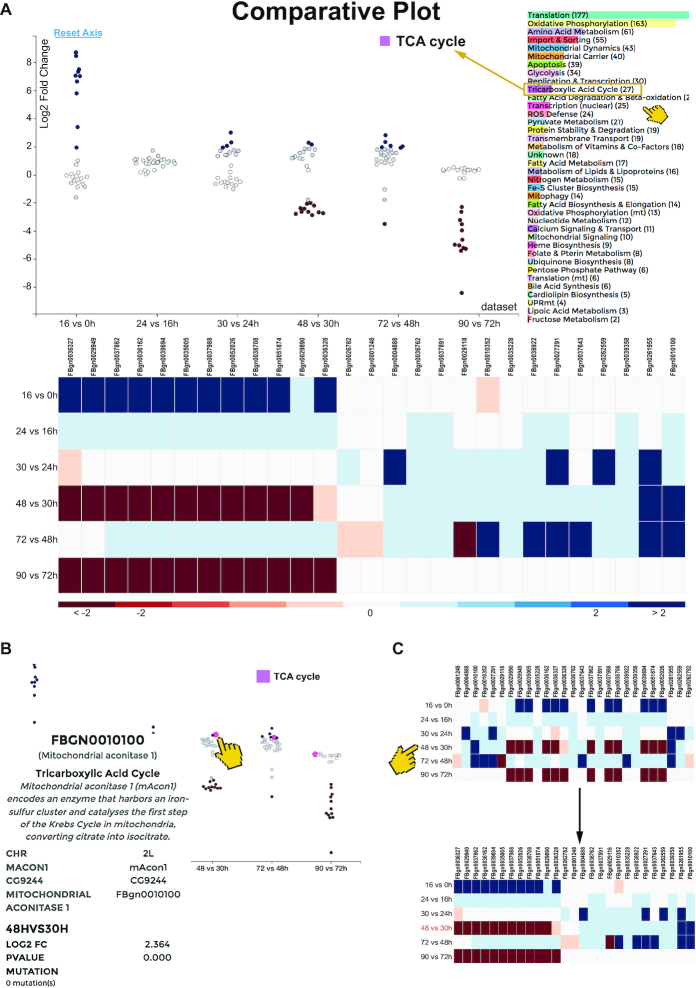
Comparative Plot of the mitoXplorer platform. (**A**) The Comparative Plot display is composed of a scatterplot, sortable heatmap and a bar chart for the selection of mito-processes. The scatterplot shows the log_2_ fold change (y-axis) and the datasets (x-axis). Each bubble represents one gene, whereby red bubbles indicate down-regulated, and blue ones upregulated genes. The process to be shown can be selected by clicking on the process name in the bar chart next to the scatterplot, the chosen process being indicated on its top. In this case, TCA cycle was chosen. The heatmap at the bottom shows the individual genes and the datasets, whereby the genes are colored according to their log_2_ fold change (indicated at the bottom of the plot). (**B**) Hovering over a gene bubble (or a gene tile in the heatmap) will display available information (in case of fly: gene name, mitochondrial process, gene description, chromosomal location, gene symbol, as well as log_2_ fold change, *P*-value and observed mutations). (**C**) The heatmap is sortable by log_2_ fold change (as indicated by the pointer in C), as well as by dataset. Clicking on one of the datasets will sort the heatmap according to the log_2_ fold change of all genes in this dataset, as is illustrated here. Clicking on one of the genes will sort the heatmap according to its log_2_ fold changes across different datasets. The time-series study of developing flight muscle (44) was used to demonstrate the functionality of this visualization method.

The Comparative Plot visualization combines several interactive graphs to analyze one mito-process, allowing the comparison of up to six datasets. It includes a scatterplot with a dynamic *y*-axis, as well as an interactive heatmap at the bottom of the page. The mito-process to be visualized can be selected in the *process panel* (Figure 3A). Red and blue coloring of the dots and the heatmap indicates the directionality of differential expression (blue: upregulated; red: down-regulated); bright blue, larger gene bubbles in the scatterplot indicate mutations, if available from the dataset. This Comparative Plot offers an overview of the expression dynamics of all members of one mito-process for up to six individual datasets and thus can be helpful in identifying co-regulated genes e.g. in time-course data, patients or multiple mutant datasets.

Hovering over a gene bubble, or over a tile in the heatmap will again display the respective associated information of the gene in the *information panel* (gene name, function, mito-process, log_2_FC, *P*-value, potential mutations) (Figure 3B). The heatmap can be sorted according to the dataset, as well as the differential expression values within one dataset (Figure 3C). The Comparative Plot is especially useful for performing a detailed, comparative, mito-process based analysis of differential expression dynamics between different datasets.

We applied this analysis method to visualize differential expression data from a time-series study of flight muscle development during pupal stages in *Drosophila* (44) (Figure 3). While enrichment analysis has revealed a general positive enrichment of processes like Tricarboxylic acid cycle (TCA Cycle) in the course of flight muscle development, mitoXplorer identifies 12 genes of TCA cycle that are co-regulated. This group of genes is strongly upregulated between 0 and 16 h after puparium formation (APF), when myoblasts divide and fuse to myotubes. The same group of genes is consecutively down-regulated in two phases at time-points 30–48 h and 72–90 h APF, when myotubes differentiate to mature muscle fibers. This is surprising as in mature muscle fibers the TCA cycle should be important for proper functioning. Their strong induction between the first two time-points could be responsible for downregulation at later stages.

## THE HEATMAP: HIERARCHICAL CLUSTERING

**Figure 4.**
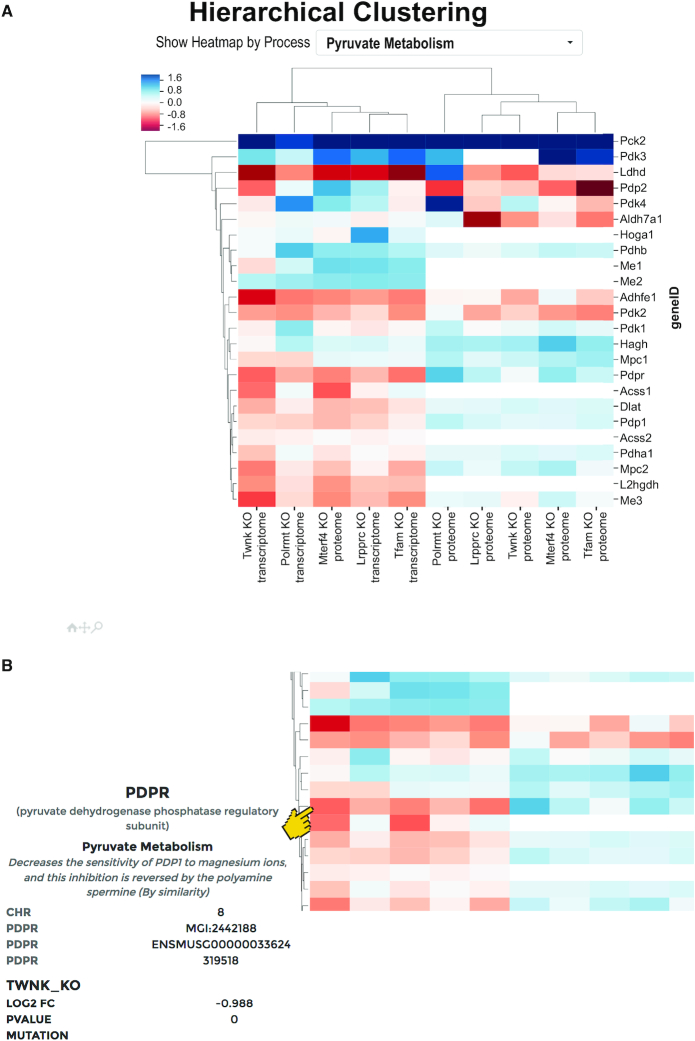
Hierarchical Clustering and heatmap plot of the mitoXplorer platform. Hierarchical Clustering of expression data results in a so-called heatmap. (**A**) Heatmap of transcriptome and proteome data of mouse knock-out strains of genes involved in mitochondrial replication, DNA-maintenance, transcription and RNA processing (taken from (40)). Data are clustered according to genes, as well as datasets. Gene tiles are colored according to their log_2_ fold change. At the top of the heatmap, the user can choose the mito-process to be displayed. (**B**) Hovering over one of the gene tiles will display information on the gene, such as the gene name, mito-process, log_2_ fold change, *P*-value and – if available – observed mutations. The heatmap is also zoom-able by clicking on the magnification glass at the bottom of the plot, so that large datasets can be visualized and analyzed efficiently. Datasets can be selected within the heatmap by grouping. To do this, first a group name has to be defined; second, the datasets belonging to this group have to be selected by clicking on one of the gene boxes of the dataset. This process can be repeated and the resulting groups can then be analyzed using Comparative Plots (see Supplementary Figure S2).

Hierarchical Clustering visualization allows the analysis of up to 100 datasets, analyzing one process at a time. This creates a heatmap with mito-genes, as well as -datasets, which are clustered according to the log_2_FC using hierarchical clustering (Figure 4 a). The results are displayed as a clustered heatmap, with a dendrogram indicating the distance between datasets or between genes.

Hovering over a gene will display its associated information, as well as dataset information in the *information panel* (Figure 4B). The user can furthermore zoom into parts of the heatmap to get a more detailed view of the data. The heatmap is particularly useful for discovering groups of similarly regulated mito-genes or datasets within one mito-process.

We applied this visualization tool to display transcriptome and proteome data from a recent, systematic study of mouse conditional knock-out strains for five genes involved in mitochondrial replication (*Twinkle (Twnk)*), mtDNA maintenance (*Tfam*), mito-transcription (*Polrmt*), mito-mRNA maturation (*Lrpprc*) and mito-translation (*mTerf4*) (40). Interestingly, the expression dynamics of the mitochondrial transcriptomes and proteomes in heart tissue did not cluster together for the mutants, suggesting strong post-transcriptional effects or protein stability changes of mito-proteins upon the loss of any of these genes. In accordance with this, the expression of some mito-genes in the process Pyruvate metabolism that is shown here differs on transcriptome and proteome level. This demonstrates the usefulness of hierarchical clustering and the heatmap display in identifying the correlation or divergence between genes as well as datasets.

## PRINCIPAL COMPONENT ANALYSIS

**Figure 5.**
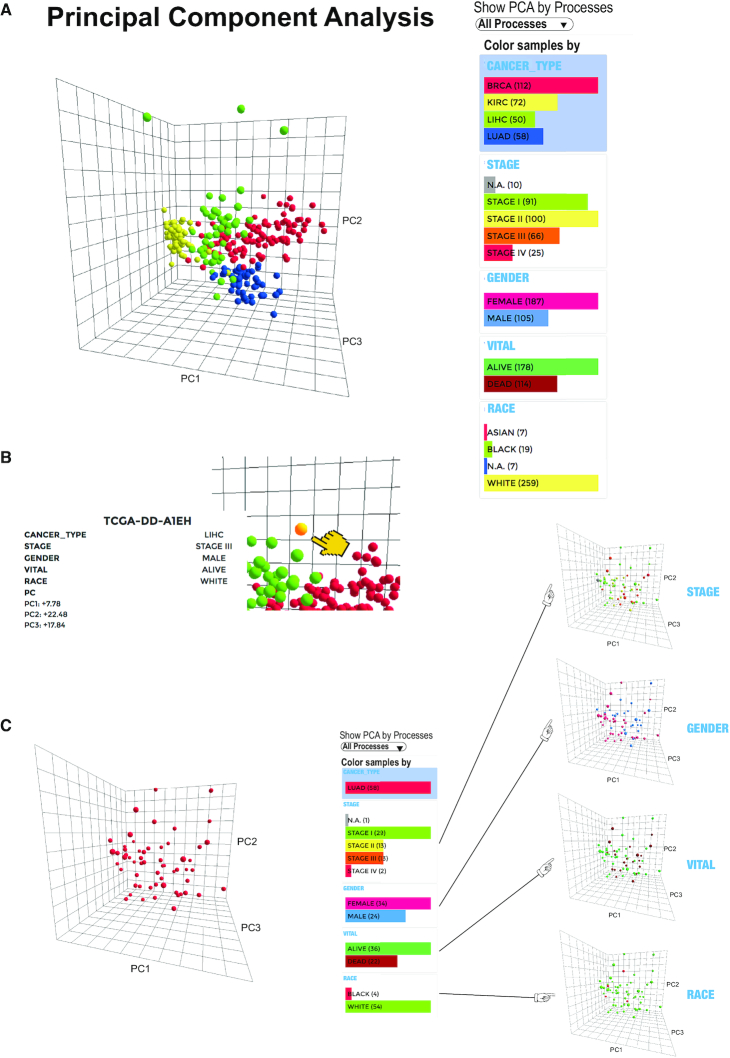
Principal component analysis and PCA plot of the mitoXplorer platform. (**A**) PCA analysis and plot of transcriptome data of The Cancer Genome Atlas (TCGA) database (1), showing four different cancer types: breast cancer (BRCA), kidney cancer (KIRK), liver cancer (LIHC) and lung cancer (LUAD). Each bubble represents one dataset, in this case, one cancer patient. At the right side at the top of the plot, the mito-process to be shown can be selected. In this case, ‘All Processes’ were selected, containing data from all mito-genes. At the right side next to the plot, different colors, as well as filters can be chosen. In this case, the Cancer Type was chosen for coloring, showing the four different cancer types in four different colors. (**B**) Hovering over a bubble will display associated information on the dataset, including the dataset name, and in case of the TCGA, information on the cancer type, the stage, the gender, the vital status, as well as skin color. In addition, the three PC components are shown. (**C**) Selecting color schemes on the right-hand side will change the coloring of the bubbles. In this case, only lung cancer is shown, and coloring is done according to Stage, Gender, Vital, and Skin color. This panel can also be used for selecting specific datasets. For instance, clicking on one of the stages will only display the chosen stage and omit datasets from other stages. As in the heatmap, datasets can be selected from the PCA for grouping. To do this, first a group name has to be defined; second, the datasets belonging to this group have to be selected by clicking on one of the dataset bubbles. This process can be repeated and the resulting groups can then be analyzed using Comparative Plots (see Supplementary Figure S2).

A larger number of datasets can be compared using Principal Component Analysis (PCA), either for an individual mito-process, or considering all mito-genes together (Figure 5A). In PCA, the expression value (e.g. log_2_FC) of each gene is considered as one dimension, and each dataset represents one data point. In the resulting 3D PCA plot, the three axes represent the first three principal components and each bubble represents one dataset. The PCA is again interactive. Mito-processes can be selected via a drop-down menu on the top of the page. The plot can be turned and moved in 3D and has a zooming function.

Hovering over a bubble will give the information associated with an individual dataset in the *information panel*, including the values of the first three principal components (Figure 5B). The information differs for each project chosen.

Individual datasets can be selected and colored via the *dataset panel* next to the plot (Figure 5C). For instance with data from TCGA the filter and coloring can be used to highlight or to limit the plot to data from different tumors, different tumor stages or according to any other additional information provided. The PCA is especially useful for analyzing a large number of datasets and displaying specific trends in sub-groups.

We used the PCA plot to visualize data from the TCGA for four cancer types stored in mitoXplorer in Figure 5A, whereby the colors of the bubbles represent the different tumor types. The plot clearly highlights the variance of the different tumor types. In particular, kidney and liver cancer are highly distinct with respect to the first three components of all mito-genes (Figure 5A).

### GROUPS function

In order to allow a more detailed, gene-centered analysis of correlated datasets, we added the possibility to select and group datasets in the Heatmap and the PCA views. Groups of datasets can be compared against each other with the Comparative Plot, whereby the log_2_FC is averaged over the data within a group. This functionality is useful, for instance when different groups of donors with similar expression patterns should be compared to each other.

We demonstrate the usability of the GROUPS function in Supplementary Figure S2, where we analyzed the averaged expression patterns of ageing human fibroblasts from healthy donors from 1 to 94 years of age (48). A first analysis using the PCA plot revealed that individuals which are older than 80 years were separated from the rest of the donors (Supplementary Figure S2A).

We next applied the GROUPS function to analyze different age groups using the mitoXplorer Comparative Plot. We chose to group individuals based on age, whereby we generated six age groups from age 40 to 100 years. As the age group 80–90 years showed two distinct clusters, we split this group in individuals that cluster with younger donors (g1), and those that cluster with the age group over 90 (g2). Our analysis using mitoXplorer GROUPS helped reveal a strong downregulation of a substantial number of mito-genes in nearly all mito-processes starting from the age of 85 (Supplementary Figure S2B), suggesting a general mitochondrial decline in old age.

Taken together, mitoXplorer provides a versatile, interactive and integrative set of tools to visualize and analyze the expression dynamics as well as mutations of mito-genes and mito-processes, facilitating a detailed understanding of observed changes at a molecular level.

### Analyzing transcriptomes of mitochondria-associated health conditions using mitoXplorer

To demonstrate the analytical and predictive power of mitoXplorer, we explored available transcriptome data from health conditions associated with mitochondrial functions. We first performed mitoXplorer analysis of data from a mouse model of Barth syndrome (46), a mitochondrial disorder caused by mutations of the Taz gene which encodes the protein Tafazzin. Second, to show that we could verify mitoXplorer predictions experimentally, we analyzed our set of trisomy 21 data using mitoXplorer (51,52).

### Analyzing the effects of Tafazzin loss of function in normoxic and hypoxic conditions using mitoXplorer

The Taz gene which encodes the cardiolipin acyl transferase Tafazzin is required for the remodeling of cardiolipin, an essential lipid component of the mitochondrial inner membrane (79). Loss of Tafazzin function leads to an abnormal fatty acid composition and a decrease in cardiolipin levels, resulting in abnormal mitochondrial morphology and dynamics, decreased stability of respiratory supercomplexes and increased oxidative stress (see (80) and references therein). Loss of Tafazzin function is also the primary cause of Barth syndrome (73), a rare, recessive, X-linked disorder that is characterized by cardiomyopathy, skeletal myopathy, growth retardation and neutropenia (81). Cardiolipin has been implicated in many mitochondrial processes, including mitochondrial protein import, mitochondrial carrier function, mitochondrial morphology and dynamics, respiratory chain function and metabolism (see (82) and references therein). Chowdhury *et al.* (46) subjected Tafazzin-mutant mouse embryonic fibroblasts (MEFs) to hypoxic stress to unravel the mechanism of impaired hypoxia-response in this cellular model of Barth syndrome. They observed that in hypoxia, a reduction of ROS levels in Tafazzin-deficient cells prohibited the induction of the NF-κB pathway, resulting in reduced Hif1α expression levels and subsequently the inability to respond to hypoxia.

**Figure 6.**
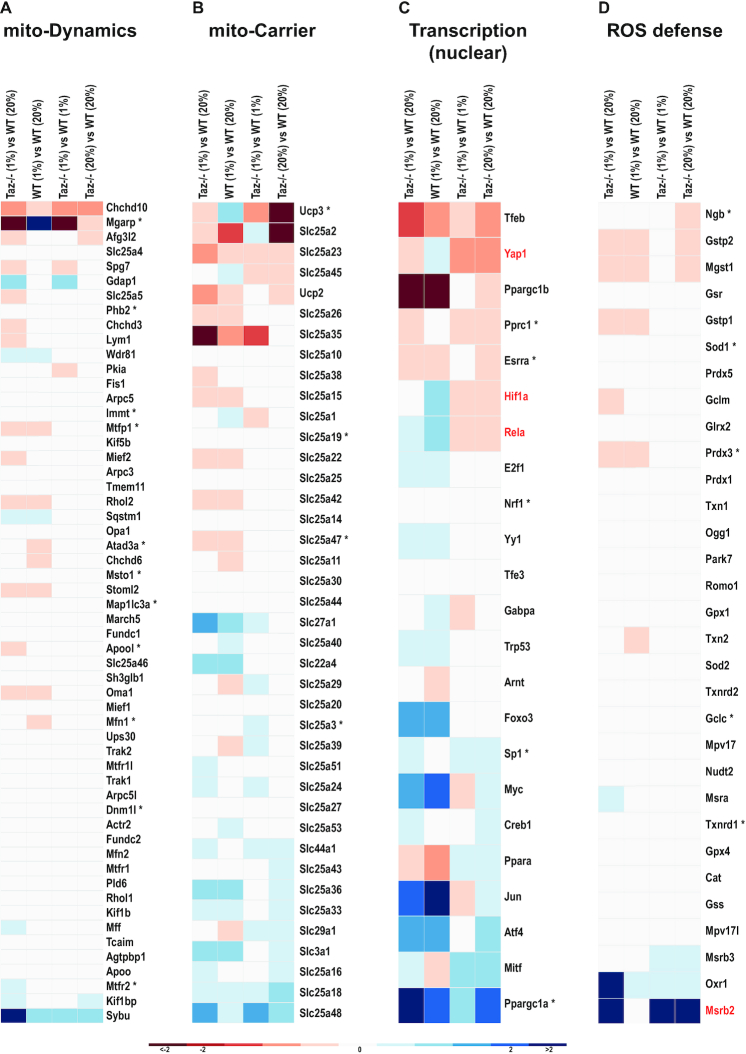
Mito-gene expression dynamics of Tafazzin-deficient mouse embryonic fibroblasts in normoxic and hypoxic conditions. The mito-processes Mitochondrial dynamics (mito-Dynamics), Mitochondrial carrier (mito-Carrier), Transcription (nuclear) and ROS defense are shown. (**A**) Among the genes involved in mito-Dynamics, the predicted Hif1α target Mgarp is strongly down-regulated in Tafazzin-deficient cells and fails to be induced in hypoxia. Mgarp is required for movement of mitochondria along microtubules during hypoxia (126). (**B**) The mitochondrial carriers Ucp3, a predicted Hif1α target, as well as Slc25a45 are differentially regulated in Tafazzin-deficient cells in hypoxia. (**C**) The transcription factors Hif1α, Yap1 (Hippo pathway) and Rela (NF-κB pathway)—all highlighted in red—are differentially regulated in Tafazzin knock-out cells in hypoxia compared to wild-type: they are induced in hypoxia and are down-regulated in Tafazzin-mutant cells in normoxic, as well as hypoxic conditions. Mitf and Ppara show opposite expression behavior, being induced in Tafazzin-deficient cells and down-regulated in hypoxia in wild-type cells. (**D**) The ROS defense gene MsrB2 (marked in red) is strongly induced in Tafazzin-deficient cells, while it remains constant in hypoxia in wild-type conditions. MsrB2 has ROS quenching properties.

Using the mitoXplorer Interactome View, we first could show that loss of Tafazzin function leads to a substantial perturbation of mito-gene expression in normoxic conditions (Supplementary Figure S3A), among which are the mito-processes Mitochondrial dynamics and Mitochondrial carrier (Figure 6A, B). In hypoxia, the expression profile of Tafazzin-deficient MEFs showed markedly different expression dynamics from wild-type cells (Supplementary Figure S3B).

In agreement with the findings from Chowdhury *et al.*, using mitoXplorer we found Hif1α down-regulated in Taz-deficient cells under normoxic and hypoxic conditions, while it was induced in hypoxia in wild-type cells (Figure 6C). Using Harmonizome (83), we also found a number of predicted and verified Hif1α target genes, some of which were de-regulated under hypoxic conditions in the Tafazzin-deficient cells (marked by an asterisk in Figure 6A–D). Next to Hif1α we found Yap1 de-regulated with the same expression dynamics. Yap1 is part of the Hippo signaling pathway and was shown to be stimulated in hypoxia (84), where it binds to and stabilizes Hif1α. As shown in Figure 6C, Yap1 was down-regulated upon loss of Tafazzin function in normoxic and hypoxic conditions, while it was upregulated in hypoxia in wild-type cells.

mitoXplorer analysis also revealed the failure of inducing RelA in hypoxia in Tafazzin-deficient cells. This transcription factor is a member of the Nf-κB family and was shown to be induced and activated early in hypoxia to modulate NF-κB target gene expression. The NF-κB dependent response to hypoxia was equally impaired upon loss of Tafazzin function.

Chowdhury and colleagues proposed that reduced ROS levels in response to hypoxia prevent proper NF-κB activation in Tafazzin-deficient cells. We thus investigated the expression dynamics of mito-genes in ROS defense using mitoXplorer and found that the Mitochondrial Methionine Sulfoxide Reductase B2 (MsrB2) was strongly induced in Tafazzin-deficient MEFs (log_2_FC of 6.67 in normoxic and 5.13 in hypoxic conditions, respectively), whereas MsrB2 levels remained constant in wild-type cells in hypoxia (Figure 6D). MsrB2 reduces methionine (R)-sulfoxide to methionine and thus decreases reactive oxygen species in the cell due to its quenching properties; thus, its overexpression protects cells from oxidative stress (85). Its strong induction could be responsible for reduced ROS levels observed in Tafazzin-deficient cells.

In conclusion, using mitoXplorer we could not only identify previously described mito-processes affected by loss of Taffazin in normoxic conditions and confirm expression changes of Hif1α in Tafazzin-deficient cells in hypoxia. The expression profiles revealed by mitoXplorer analysis furthermore suggest that loss of Yap1 contributes to the observed phenotype by de-stabilizing Hif1α. Moreover, our analysis using mitoXplorer indicates that massive induction of the MsrB2 gene could be responsible for reduced ROS levels in Tafazzin-deficient cells in hypoxia, leading to a failure of induction of the NF-κB pathway and the transcription factor RelA.

### Analyzing cell lines carrying trisomy 21 using the mitoXplorer platform

We next wanted to experimentally verify predictions made with mitoXplorer. To this end, we analyzed the transcriptome and proteome of a set of aneuploid cell lines carrying an extra copy of chromosome 21 (trisomy 21, T21). Mitochondrial dysfunction has been repeatedly found in T21 patients, whereby mostly oxidative stress, as well as—potentially resulting—mitochondrial respiratory deficiency have been shown to contribute to some of the observed clinical features (see for instance (86–99)). Transcriptome studies of different T21 tissues using microarrays (100–110) and more recently RNA sequencing (42,43,111) and proteomics (41,112–115) have revealed a complex picture of gene expression changes, with a marked dissimilarity in differential expression of mito-genes on mRNA and protein levels, indicating a potential post-transcriptional regulatory effect of some mito-genes in T21 (41). Yet, mito-gene and protein expression data in different tissues or under varying conditions in T21 remain sparse and a coherent hypothesis of the underlying mechanisms leading to the mitochondrial deficiencies in T21 patients is still missing.

We used trisomy 21 cell lines derived from either the euploid human colon cancer cell line HCT116 or from the retinal pigmented epithelial cell line RPE1, to which an extra copy of chromosome 21 was added (51). We used two RPE1-derived and two HCT116-derived clones trisomic for chromosome 21 (Supplementary Table S3 a), which were validated by fluorescent *in situ* hybridization and by whole genome sequencing. We used transcriptomic data of the original euploid RPE1 line and its two trisomic derivatives (RPE_T21 clone 1 and 2 (c1, c2) (51)), as well as for HCT116, and its trisomic derivatives (HCT_T21 (c1, c3)). We included proteomic data for RPE1 and one of its T21 derivatives (RPE_T21 c1). We performed bioinformatic analysis to determine differential expression of the above conditions (Supplementary Table S3B–E, for details on bioinformatic data analysis see Materials and Methods) and uploaded the differential expression data of the transcriptome and proteome on the mitoXplorer platform for further in-depth, mitochondrial analysis.

### Differences between trisomy 21 cell lines

**Figure 7.**
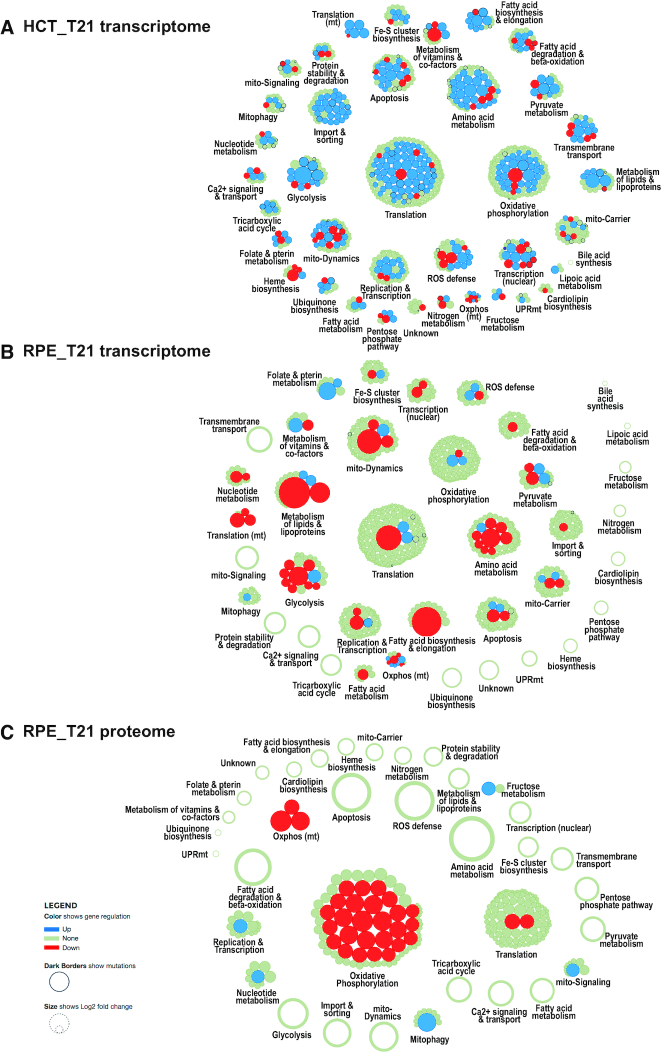
Interactome View of the transcriptome and proteome of cell lines carrying trisomy 21. Trisomy 21 samples were compared against their wild-type counterpart. Transcriptomic analysis of (**A**) HCT116_T21 (trisomy 21 against wild-type, c3) and (**B**) RPE21_T21 (trisomic against wild-type, c1); (**C**) proteomic analysis of RPE_T21 cells (trisomy 21 against wild-type, c1). Transcriptome changes are different between the two trisomy 21 cell lines HCT116 and RPE1. Expression changes of HCT_T21 cells are mild and genes tend to be upregulated (**A**), while some genes are strongly down-regulated in RPE_T21 cells (**B**). The transcriptome (**B**) and the proteome (**C**) of RPE_T21 cells respond quite differently, with a strong downregulation of components of the process Oxidative phosphorylation (OXPHOS) at proteome level which is not observed on transcriptome level. Most genes differentially expressed at transcript level, on the other hand, show no significant changes on proteome level. Red bubbles indicate downregulation, blue ones indicate upregulated genes. The size of the bubble corresponds to the log_2_ fold change.

MitoXplorer analysis of data comparing HCT116- and RPE1-derived T21 cell lines using the Interactome View revealed that T21 induced strong effects with respect to the overall expression changes in mito-genes (Figure 7). HCT_T21 showed a subtle, but consistent up-regulation of mito-genes (Figure 7A). In contrast, RPE_T21 cells showed a strong downregulation of a few genes involved in several mito-processes, such as Fatty acid metabolism, Glycolysis or Mitochondrial dynamics (Figure 7B). Remarkably, quantitative proteome data from RPE_T21 c1 cells suggested that all mitochondria-encoded genes involved in OXPHOS, as well as the majority of nuclear-encoded OXPHOS-genes are down-regulated (Figure 7C). In conclusion, mitoXplorer analysis facilitated the finding of significant differences in mito-gene expression between the different cell lines. Importantly in RPE_T21 cells, proteome data showed a remarkable difference to transcriptome data.

### mitoXplorer analysis suggests mitochondrial ribosomal assembly defects in RPE_T21 cell lines

**Figure 8.**
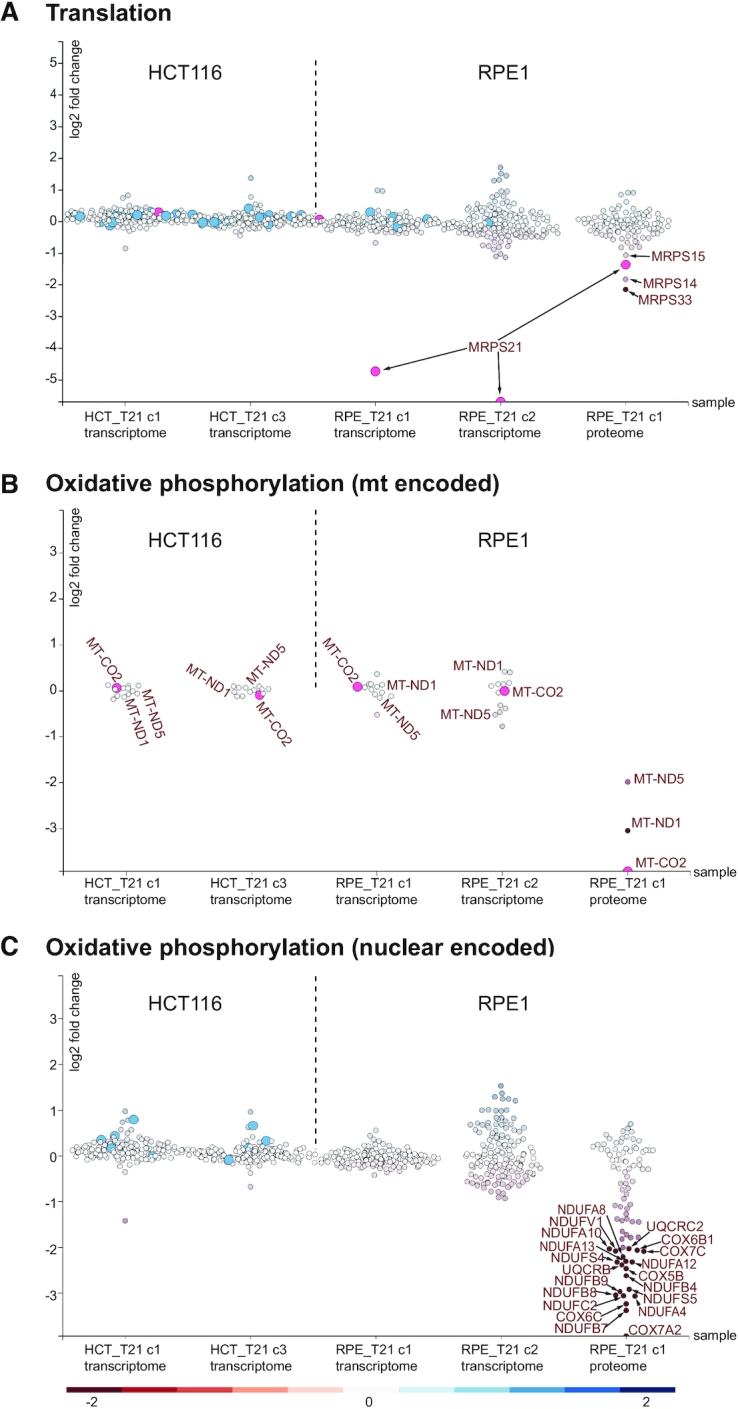
Scatterplots of Translation, mitochondrial- as well as nuclear components of Oxidative phosphorylation of trisomy 21 cells. (**A**) In the mitochondrial process Translation, MRPS21 is strongly down-regulated on transcriptome level in RPE_T21 cells as compared to RPE1 wild-type (wt) cells. No change is observed in HCT_T21 cells. On proteome level, several components of the mitoribosome small subunit (SSU) are down-regulated in RPE_T21 cells. (**B**) Transcript levels of mitochondria-encoded genes of Oxidative phosphorylation (OXPHOS) are not affected, while those genes are strongly diminished on protein level. (**C**) A significant number of components of OXPHOS are down-regulated on protein level in RPE_T21 cells, while no significant or only mild reduction can be observed on transcriptome level in trisomy 21 cell lines. Scatterplots are taken from the mitoXplorer Comparative Plot interface. Each bubble represents one gene, light blue dots indicate mutated genes. On the y-axis, the log2 fold change is plotted, the cell lines (transcriptome of HCT116 T21 (HCT_T21) clone 1 (c1) and clone 3 (c3) versus wild-type, as well as transcriptome of RPE1 T21 (RPE_T21) clone 1 (c1) and clone 2 (c2) versus wild-type and proteome of RPE1 T21 clone 1 (RPE_T21 c1) versus wild-type) are plotted on the x-axis. The gene highlighted in pink has been selected on the web-server: MRPS21 for the process Translation; MT-CO_2_ for the process Oxidative phosphorylation (mt); and no gene has been selected in the process Oxidative phosphorylation.

To investigate the differences further, we next performed a more detailed analysis of expression changes in these T21 cell lines using Comparative Plots in mitoXplorer. Transcriptome and proteome data from RPE_T21, but not from HCT_T21 cell lines revealed that several subunits of the small mitochondrial ribosome (mitoribosome) were significantly down-regulated on either RNA or protein level, or both (Figure 8A). MRPS21 was strongly reduced on RNA- and protein-level. The genes MRPS33, MRPS14 and MRPS15 were largely normal on RNA level, while their protein levels decreased more than 2-fold (log2FC: MRPS33: -2.147; MRPS14: −1.827; MRPS15: −1.057). Mitoribosomal subunits are encoded in the nuclear genome and their protein products are imported into the mitochondria, where they assemble with mitochondrial ribosomal RNAs to form the large and small subunits of the mitoribosome. The mitoribosome is responsible for translating the 13 mt-mRNAs encoded in the mitochondrial genome, all of which code for key subunits of the respiratory chain required for OXPHOS (116,117). In accordance with a disrupted mitochondrial translation machinery, all quantifiable mitochondria-encoded OXPHOS proteins (Complex I: MT-ND1 and MT-ND5; Complex IV: MT-CO2) were severely diminished on protein-, but not on RNA-level in RPE1_T21 cells (Figure 8B).

Interestingly, 36 of the quantifiable OXPHOS proteins encoded in the nuclear genome were also found to be down-regulated at the proteome, but not at transcriptome level in RPE_T21 cells (Figure 8C). These include subunits of the NADH dehydrogenase (complex I), ubiquinol−cytochrome *c* reductase (complex III) and cytochrome c oxidase (complex IV). It is important to note that there is no general downregulation of mitochondrial proteins in these cells and only a few, specific proteins are strongly down-regulated (Figure 7C). Together, these data demonstrate the power of mitoXplorer to help identify the cause of important changes in mito-gene expression, here the downregulation of mitoribosomal subunits at the transcription level and the resulting consequences, in this case the downregulation of the majority of OXPHOS proteins.

### RPE_T21 cells are defective in oxidative phosphorylation

**Figure 9.**
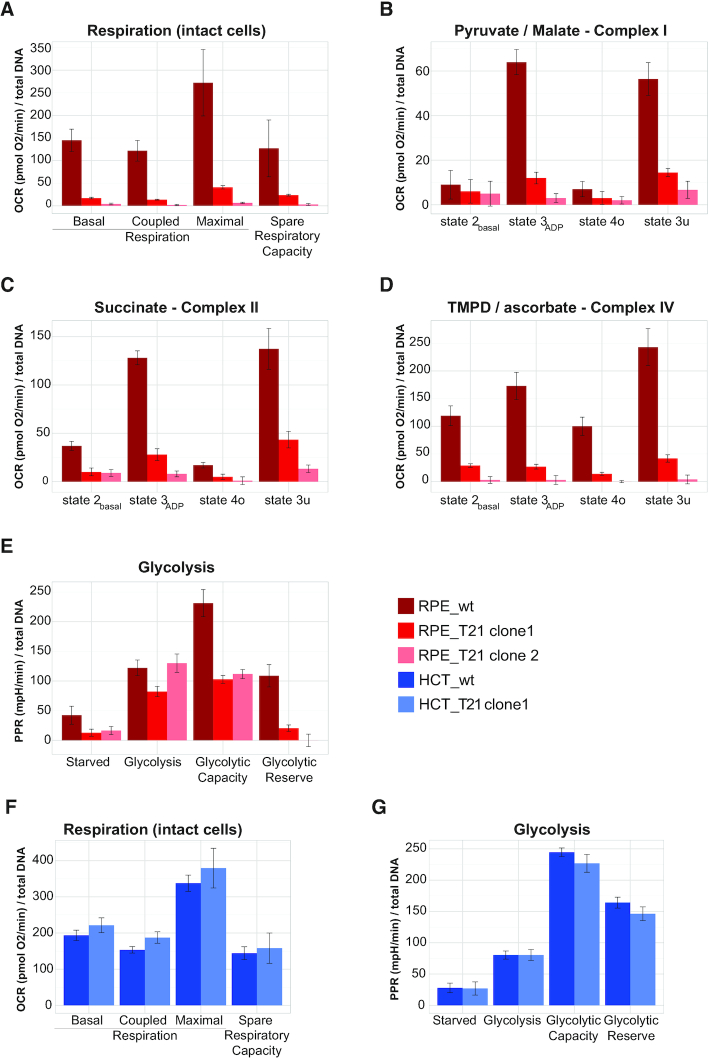
Mitochondrial respiration and glycolysis is strongly affected in RPE_T21 cells and not affected in HCT_T21 cells. (**A**) Respiration in intact RPE_T21 cells is greatly decreased compared to wild-type. (**B–D**) Permeabilized RPE_T21 cells supplemented for substrates of complex I, II and IV as indicated in the header of each plot, showed equally dysfunctional OXPHOS, suggesting a general break-down of the respiratory chain. (**E**) RPE_T21 cells do not have any spare glycolytic reserve. Respiration (**F**), as well as glycolysis (**G**) is virtually unchanged in HCT_T21 cells compared to their wild-type counterparts. Bright red: RPE_T21 clone 1; light red: RPE_T21 clone 2; dark red: RPE wild-type; dark blue: HCT wild-type; light blue: HCT_T21 clone 1. Measurements of cellular respiration in intact and permeabilized cells, as well as glycolytic potential were done using the Seahorse Bioscience XF Extracellular Flux Analyzer (Seahorse Biosciences). The experiments were performed using the mitochondrial and glycolytic stress test assay protocol as suggested by the manufacturer; the rate of cellular oxidative phosphorylation (oxygen consumption rate (OCR)) and glycolysis (cellular proton production rate (PPR)) were measured simultaneously.

The massive downregulation of OXPHOS proteins in RPE_T21 cells suggests that these cells should suffer from a severe OXPHOS deficiency. To test this hypothesis experimentally, we analyzed cellular respiration and glycolysis in T21 cell lines using a Seahorse XF96 analyzer to quantify oxygen consumption rate (OCR) as an indicator of mitochondrial respiration (Figure 9A−D, F), as well as the proton production rate (PPR) as an indicator of glycolysis (Figure 9E, G). In intact RPE_T21 cells, we indeed observed dramatically reduced levels of cellular respiration in comparison to the diploid control (Figure 9A).

As a complex I deficiency has been reported in trisomy 21 patients (95), we next asked whether RPE_T21 cells selectively suffer from a complex I deficiency, or whether the entire respiratory chain is affected, as suggested by our proteomic data. We used permeabilized cells to test each individual complex with the Seahorse analyzer, supplementing with pyruvate/malate, succinate and TMPD/ascorbate for assessing complex I, II or IV functionality, respectively. As expected from our proteomic analysis, RPE_T21 cells displayed a severe deficiency of the entire respiratory chain (Figure 9B−D). The glycolytic rate of RPE_T21 cells in the presence of glucose was similar to the diploid control cells. Inhibition of ATP-production was not able to stimulate the cells to a higher glycolytic rate (Figure 9E), which agrees with the already low OXPHOS levels observed in these cells. HCT_T21 cells, on the other hand, displayed normal respiration, as well as glycolysis (Figure 9F, G). This suggests that the respiratory chain, as well as the mitochondrial translational machinery is not generally affected in all T21 cells. Taken together, mitoXplorer helped uncover OXPHOS deficiencies in RPE_T21 cells, which we verified experimentally, demonstrating the power of an in-depth analysis of mitochondrial expression dynamics to identify the potential molecular cause of the observed phenotype.

### Quantification of mitochondrial network morphology using mitoMorph

**Figure 10.**
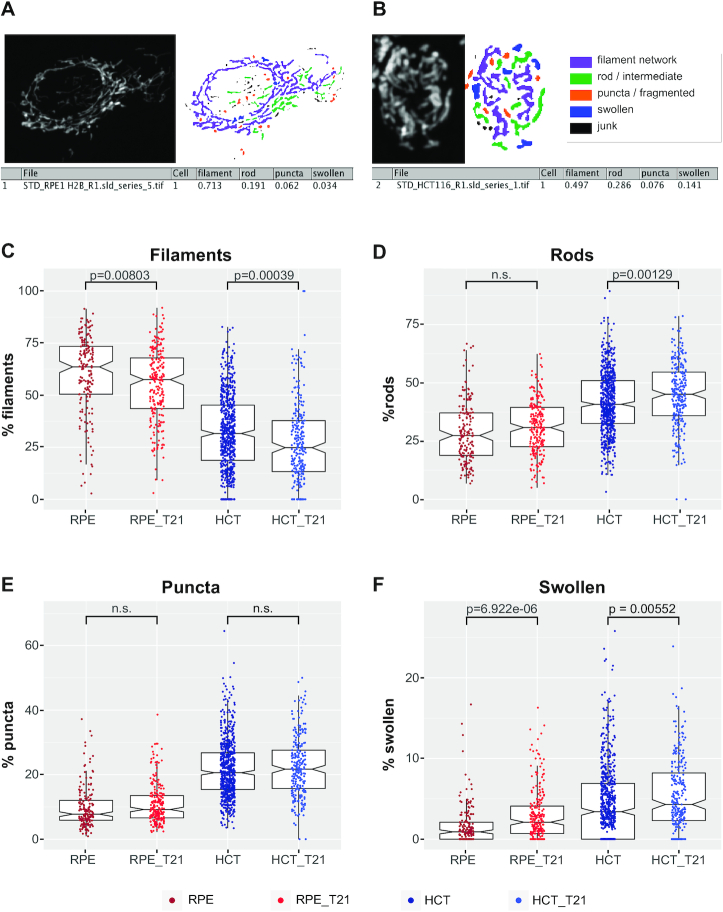
Mitochondrial morphology is slightly changed in trisomy 21 cells. We have stained the mitochondrial network and analyzed the network morphology, measuring the percentage of filamentous networked (filaments), rods, puncta and swollen using the Fiji plugin mitoMorph. (**A**) Sample of mitoMorph analysis of an RPE1 wild-type cell and (**B**) of an HCT116 wild-type cell. Filamentous networks are highlighted in lilac, rods in green, puncta (referring to fragmented mitochondria) are highlighted in orange and swollen ones in blue. The percentage filaments, rods, puncta and swollen mitochondria are automatically scored and reported to the user. (**C**) the percentage of filaments is slightly, but significantly reduced in RPE_T21, as well as HCT_T21 cells compared to wild-type. (**D**) There is no significant change in the percentage of rods in RPE_T21 cells and slightly higher percentage in HCT_T21 cells compared to wild-type. (**E**) The percentage of puncta is unchanged in both T21 cell lines. (**F**) There are significantly more swollen mitochondria in both, RPE_T21, as well as HCT_T21 cells compared to wild-type. Results have been averaged over both clones of each cell line, respectively. Underlying numerical values are provided in Supplementary Table S1, sample images are shown in Supplementary Figure S7.

We further wanted to investigate, if T21 and the defective OXPHOS had a consequence on mitochondrial morphology and the mitochondrial network structure was changed in T21 cell lines. To quantify mitochondrial morphology in RPE_T21 cells, we stained mitochondria using the MitoTracker Deep Red FM dye. In order to quantify the characteristics of mitochondrial morphology, we developed a new Fiji plugin for quantification of mitochondrial network features, which we called mitoMorph. MitoMorph is based on the scripts provided by Leonard *et al.* (60) for quantifying mitochondrial network features such as *filaments* (corresponding to filamentous networked structures longer than 11 μm), *rods* (corresponding to filamentous networked structures shorter than 11 μm), *puncta* (corresponding to round structures below a radius of 0.6 μm) and *swollen* (corresponding to round structures above a radius of 0.6 μm) mitochondrial structures (see Methods for implementation details). MitoMorph reports the percentages of filaments, rods, puncta and swollen for each individual cell, as well as for all selected cells in a batch analysis (Figure 10A, B). Moreover, it provides the lengths and areas of filaments and rods. Figure 10C−F shows the distribution of mitochondrial network features for the two wild-type and T21 cell lines. MitoMorph analysis revealed that in both backgrounds, T21 cells had fewer mitochondrial filaments than their wild-type counterparts, but instead possessed a slightly higher number of rods, which was significant in HCT_T21 cells. Both T21 cell lines had significantly more swollen structures than their wild-type counterparts. Length and area distribution of filaments and rods were not significantly different between the wild-type and the trisomy 21 cells (Supplementary Figure S4A−D). We looked at expression dynamics of mito-genes associated with the process Mitochondrial dynamics using mitoXplorer. The only gene that is consistently, though only mildly down-regulated in both RPE_T21 clones is GDAP1 (Supplementary Figure S5). GDAP1 was shown to regulate the mitochondrial network by promoting mitochondrial fission (118). Its downregulation could be contributing to or be a consequence of the observed phenotype. In conclusion, mitochondrial morphology based on light-microscopy is mildly affected in trisomy 21.

### Data integration with publicly available trisomy 21 datasets

After discovering this differential OXPHOS defect in our RPE_T21 cell lines, we were interested in the overlap of the mito-transcriptome and -proteome of RPE_T21 cells with data from trisomy 21 patients. We used proteomic and transcriptomic data from a monozygotic twin study discordant for chromosome 21 (41,42). In agreement with our RPE_T21 data, systematic proteome and proteostasis profiling of fibroblasts from monozygotic twins discordant for T21 revealed a significant, although milder downregulation of the mitochondrial proteome, including proteins involved in OXPHOS, which is not apparent from transcriptomic analysis of the same cells (see Supplementary Figure S6A, B).

We next looked at proteomic data of fibroblasts from 11 unrelated individuals with trisomy 21 (41) (Supplementary Figure S6C−E). Virtually all T21 patients showed reduction in at least a few mitochondrial- and nuclear-encoded subunits of the respiratory chain (Supplementary Figure S6D, E). However, we could not confirm the strong reduction of the MRPS21 protein in all individuals. The only measurable mitoribosome subunit that was consistently, though in some cases only mildly, down-regulated was MRPL19 (Supplementary Figure S6C). Taken together, though the precise molecular mechanisms remain elusive, our analysis of these datasets with mitoXplorer nevertheless suggests a post-transcriptional effect leading to reduced expression levels of proteins involved in OXPHOS in trisomy 21.

## DISCUSSION

### The web-based mitoXplorer platform for mito-centric data exploration

MitoXplorer is a practical web tool with an intuitive interface for users who wish to gain insight from -omics data in mitochondrial functions. It is the first tool that takes advantage of the breadth of -omics data available to date to explore expression variability of mito-genes and -processes. It does so by integrating a hand-curated, annotated mitochondrial interactome with -omics data available in public databases or provided by the user.

MitoXplorer has been conceived and implemented as a visual data mining (VDM) platform: by iteratively interacting, visualizing and by allowing manipulation of the graphical display of data, the user can effectively discover complex data to extract knowledge and gain deeper understanding of the data. MitoXplorer provides a set of particularly interactive and flexible visualization tools, with a fine-grained, function- as well as gene-based resolution of the data. Clustering, as well as PCA-analysis help in addition to mine a larger number of -omics data effectively by grouping datasets with similar expression patterns.

VDM-based knowledge discovery is offered by a large number of resources and platforms. However, to the best of our knowledge, no currently available tool allows to explore expression variation of a specific subset of genes in a large number of -omics datasets. It permits users to exploit publicly available transcriptome, proteome or mutation data to study the variation and thus, the adaptability of a defined gene set in different conditions or species. While mitoXplorer offers the exploration of mito-genes, we have designed the platform in such a way that users interested in a different gene group can download a local version of mitoXplorer and upload their own interactome, which may contain any gene group of interest. Thus, mitoXplorer can be flexibly adjusted to any user-defined gene set.

### Identification of putative causes of ROS-downregulation in Tafazzin-deficient cells using mitoXplorer

We have analyzed data from a mouse model of Barth syndrome to demonstrate that mitoXplorer can help identify de-regulated pathways in mitochondria-associated diseases. Barth syndrome results from a dis-balance of cardiolipin species due to the loss-of-function of the Tafazzin protein and displays defects in many mitochondrial processes. Chowdhury *et al.* have specifically tested the response to hypoxia of Tafazzin-deficient cells. They have identified reduced ROS levels in Tafazzin-deficient MEFs that lead to a failure to induce the NF-κB transcription factor RelA and finally, the transcription factor Hif1α. Using the more comprehensive and manually curated mito-interactome of mitoXplorer, we could not only confirm the failure of induction of RelA and Hif1α. We furthermore propose that failed induction of the Hippo pathway protein Yap1 which stabilizes Hif1α could contribute to the observed phenotype. Finally, using mitoXplorer we identified MrsB2 as the putative cause of lowered ROS levels in Tafazzin-deficient cells, as this enzyme has ROS-quenching activities, normally protecting cells from oxidative damage.

### Cell type-specific de-regulation of mito-genes in trisomy 21

We experimentally verified mitoXplorer predictions by expression profiling of mito-genes in T21 cell lines. Mito-genes were strongly deregulated in both trisomic cell types tested, the non-cancerous retinal pigment epithelial cell line RPE1 and the cancer cell line HCT116. Yet, the changes in expression were quite different in the two cell lines. It is not unexpected that mito-genes are differentially expressed in different cell types, reflecting the divergent cellular energy- and metabolic demands (20). Gene expression is moreover tightly regulated in a cell-type specific manner by regulating transcription, translation and the epigenetic state of the cell. Thus, also divergent and cell-type specific expression changes of mito-genes upon introduction of an extra chromosome is not surprising.

### mitoXplorer assisted in revealing divergent de-regulation of mitochondrial transcriptome and proteome in trisomy 21

We found a remarkable difference between transcriptome and proteome levels of mito-genes in RPE_T21 cells. In particular the OXPHOS proteins were strongly down-regulated at protein, but not mRNA level. This can be explained by essential components of the respiratory chain being encoded in the mitochondrial genome and thus requiring a functioning mitochondrial replication system, as well as intact mitochondrial transcription and translation. Thus, there is as strong post-transcriptional regulation of the mitochondrial proteome. In case of the RPE_T21 cell line, the disintegration of the mitoribosome and thus a failure of mitochondrial translation is likely causative for the downregulation of OXPHOS components on protein-level, possibly by proteolysis, as the essential mitochondrial subunits are not produced and thus complexes cannot assemble. This conclusion is further supported by the fact that we could not observe a significant difference in mitochondrial transcript levels, with some mt-mRNAs even being upregulated; thus, mtDNA -maintenance, -replication as well as mito-transcription seem to be unaffected.

MitoXplorer analysis of previously published data of the mito-proteome of fibroblasts isolated from monozygotic twins discordant for T21, as well as 11 unrelated individuals with trisomy 21 (41) confirmed a similar post-transcriptional effect as we found in our T21 model cell lines. Taken together, our data uncovered a significant post-transcriptional regulation of the mitochondrial process OXPHOS in our model system of trisomy 21 that could bring new insight into the mechanisms of mitochondrial defects in trisomy 21 patients.

### mitoXplorer helped identify mitochondrial ribosomal protein S21 (MRPS21) as potentially causative for OXPHOS failure

The most notable difference in RPE_T21 cells compared to wild-type is the >10-fold downregulation of mitochondrial ribosomal protein S21 (MRPS21) on transcript level, as well as the downregulation of Mrps21 protein and other proteins of the small and—to a lesser extend—large mitoribosome subunits. Thus, our data suggest that the integrity of the mitoribosome is compromised, leading to its disintegration and subsequently, the downregulation of mitochondrial proteins of the respiratory chain. Mrps21 is a late-assembly component and lies at the outer rim of the body (or bottom) of the small subunit (SSU) of the mitoribosome. Nevertheless, it interacts with a number of other proteins of the SSU and also directly contacts bases of the 12S rRNA (119,120). Thus, its absence could destabilize the SSU of the mitoribosome. The two most down-regulated proteins are Mrps33 and Mrps14, both of which directly interact with each other and several other proteins in the SSU and are localized to the head of the SSU. Furthermore, together with another down-regulated component, Mrps15, they are proteins that are incorporated late in the mitoribosome assembly process (120). This raises the possibility that late-assembly proteins disintegrate more readily from the mitoribosome, leading to their enhanced degradation and thus ribosome malfunction.

Based on promoter analysis using MotifMap (121), potential binding motifs of two transcription factors located on chromosome 21, GABPA and ETS2, can be found in the promoter region of the MRPS21 gene. Gabpα, which is also known as nuclear respiratory factor 2, has already been implicated in mitochondrial biogenesis by regulating Tfb1m expression (122): its depletion in mouse embryonic fibroblasts showed reduced mitochondrial mass, ATP production, oxygen consumption and mito-protein synthesis, but had no effect on mitochondrial morphology, membrane potential or apoptosis. Direct or indirect regulation of mitoribosomal proteins could be another regulatory function of this transcription factor. GABPA is not affected on transcriptome level, but is down-regulated on protein-level in RPE_T21 cells. ETS2 on the other hand has so far not been implicated in mitochondrial biogenesis or functional regulation.

We see consistent downregulation of proteins involved in OXPHOS in other trisomy 21 proteomic datasets and OXPHOS defects have been reported in trisomy 21 before. MRPS21 seems deregulated only in a few T21 individuals. Thus, the causes of OXPHOS deficiencies seem to depend on genomic background or on the cell type studied. Trisomy 21 patients develop different degrees of severity of symptoms and it is likely that the genomic variability of chromosome 21 contributes to the varying phenotypes (123,124). In conclusion, while defects in OXPHOS seem a common phenotype in trisomy 21, their severity as well as the underlying mechanisms might differ depending on the cellular model or the genomic background.

### Limitations and future developments of mitoXplorer

The proper assembly and annotation of the mito-interactomes turned out to be a demanding task. While proteomic studies of mitochondria are available for different model species and humans, the data cannot be taken without manual intervention due to significant numbers of false-positives and false-negatives. A manual curation of the data is therefore mandatory. Though we carefully curated the mito-interactomes used in mitoXplorer, we do not claim that they are complete or free of false-positives.

We decided to assign one mito-gene with one mito-process only, as it was the most straightforward solution to implement. Furthermore, assigning multiple processes to one gene would artificially increase the mitochondrial interactome, making the analysis also more difficult. Nevertheless, the current annotation might not represent all experimentally validated biological functions of a mito-gene. In future releases, we will therefore consider allowing two or more processes for one mito-gene at least in a limited number of cases, whenever there is strong experimental evidence that a protein or protein complex is contributing to two or more mito-processes. We hope that the scientific community working on mitochondria will help us further clean, complete and correctly annotate our mito-interactomes by using the FEEDBACK page.

The current version of mitoXplorer does not provide mito-process enrichment analysis. Our reasoning behind this decision was to allow users to mine their data in an unbiased and detailed way, considering all mito-processes rather than only focusing on enriched ones. In future releases, we will consider adding information on mito-process enrichment of a dataset to guide users in their analysis, for example by visually highlighting enriched processes in the Interactome View.

MitoXplorer has been optimized for mining expression data and currently it is not meaningful to analyze mito-gene mutations alone. We realize that this is a limitation when considering large, population-wide studies. We will therefore consider implementing a visual data mining interface that is specifically tailored for analyzing mito-gene mutations alone in future versions of the software.

MitoXplorer expects users to provide data for which differential expression analysis or mutation calling has already been performed, as mitoXplorer was conceived as a visual data mining platform. There are already many tools and pipelines available to perform differential expression analysis and mutation calling, yet too few tools that allow in-depth data mining, such as mitoXplorer. To make prior data analysis as easy as possible for users, we provide a pipeline for differential expression analysis and mutant calling, which is available in our git-repository (https://gitlab.com/habermannlab/mitox_rnaseq_pipeline/). Nevertheless, we recognize that the potential heterogeneity of analyzed data from different studies has limitations, especially concerning comparative analysis between different projects.

MitoXplorer is integrating, clustering and visualizing numerical data resulting from expression studies (transcriptome, proteome), as well as mutation data. Thus, it is currently limited to analyzing mito-genes without offering the ability to explore their embedding in a broader, cellular context and thus to learn about potential regulatory mechanisms of observed expression changes of mito-genes. Therefore, in the next release of mitoXplorer, we plan to fully embed mito-genes within the cellular gene regulatory, as well as signaling network by adding information from epigenetic studies (ChIP-seq, methylation data), as well as from the cellular interactome. We will provide the tools to perform enrichment analysis of observed transcription factors binding in the promoter regions of co-regulated mito-genes. We will embed a method to analyze promoter regions of mito-genes, as we have shown here for MRPS21, or to identify targets of transcription factors as here demonstrated for Hif1α. Furthermore, we will make available network analysis methods such as viPEr (125) to explore the cellular network regulating mito-genes. Other analysis methods we plan to provide include correlation analysis, as well as cross-species data mining. Depending on user requests, we could also add the mitochondrial interactomes of other species. As mitoXplorer stores the mitochondrial interactomes and the associated -omics data in a MySQL database, all technical requirements for extending the functionalities of mitoXplorer are already implemented.

## CONCLUSIONS

mitoXplorer is a powerful, web-based visual data mining platform that allows users to in-depth analyze and visualize mutations and expression dynamics of mito-genes and mito-processes by integrating a manually curated mitochondrial interactome with -omics data in various tissues and conditions of four model species, including human. We used transcriptome and proteome data from cell lines with trisomy 21 to demonstrate the value of mitoXplorer in analyzing in detail the expression dynamics of mito-genes and -processes. We have used mitoXplorer to integrate these data with publicly available datasets of patients with trisomy 21. Using mitoXplorer for data mining, we predicted failure of mitochondrial respiration in one of the trisomy 21 cell lines, which we verified experimentally. Our results demonstrate the power of a visual data mining platform such as mitoXplorer to explore expression dynamics of a specified mito-gene set in a detailed and focused manner, leading to discovery of underlying molecular mechanisms and providing testable hypotheses for further experimental studies.

## DATA AVAILABILITY

The mitoXplorer web-server is freely available at http://mitoxplorer.ibdm.univ-mrs.fr/. The source code of mitoXplorer is available at https://gitlab.com/habermannlab/mitox. The pipeline for differential expression analysis and mutation calling of RNA-seq data is available at https://gitlab.com/habermannlab/mitox_rnaseq_pipeline. MitoMorph is freely available at https://github.com/giocard/mitoMorph. RNA-seq data published with this study are available via the Gene Expression Omnibus (GEO) database (accession number: GSE131249).

## Supplementary Material

gkz1128_Supplemental_Files
