# Supplementary material for: mitoXplorer, a visual data mining platform to systematically analyze and visualize mitochondrial expression dynamics and mutations
Source: Nucleic Acids Res. 2019 Dec 4;48(2):605–32. doi: 10.1093/nar/gkz1128 (PMC6954439; doi:10.1093/nar/gkz1128)
Supplement: gkz1128_Supplemental_Files [file gkz1128_supplemental_files.zip › Yim_etal_SupplementaryFigures_clean.pdf]

Supplementary Figures 1 – 7, Supplementary Figures Legends

Supplementary Figure S1

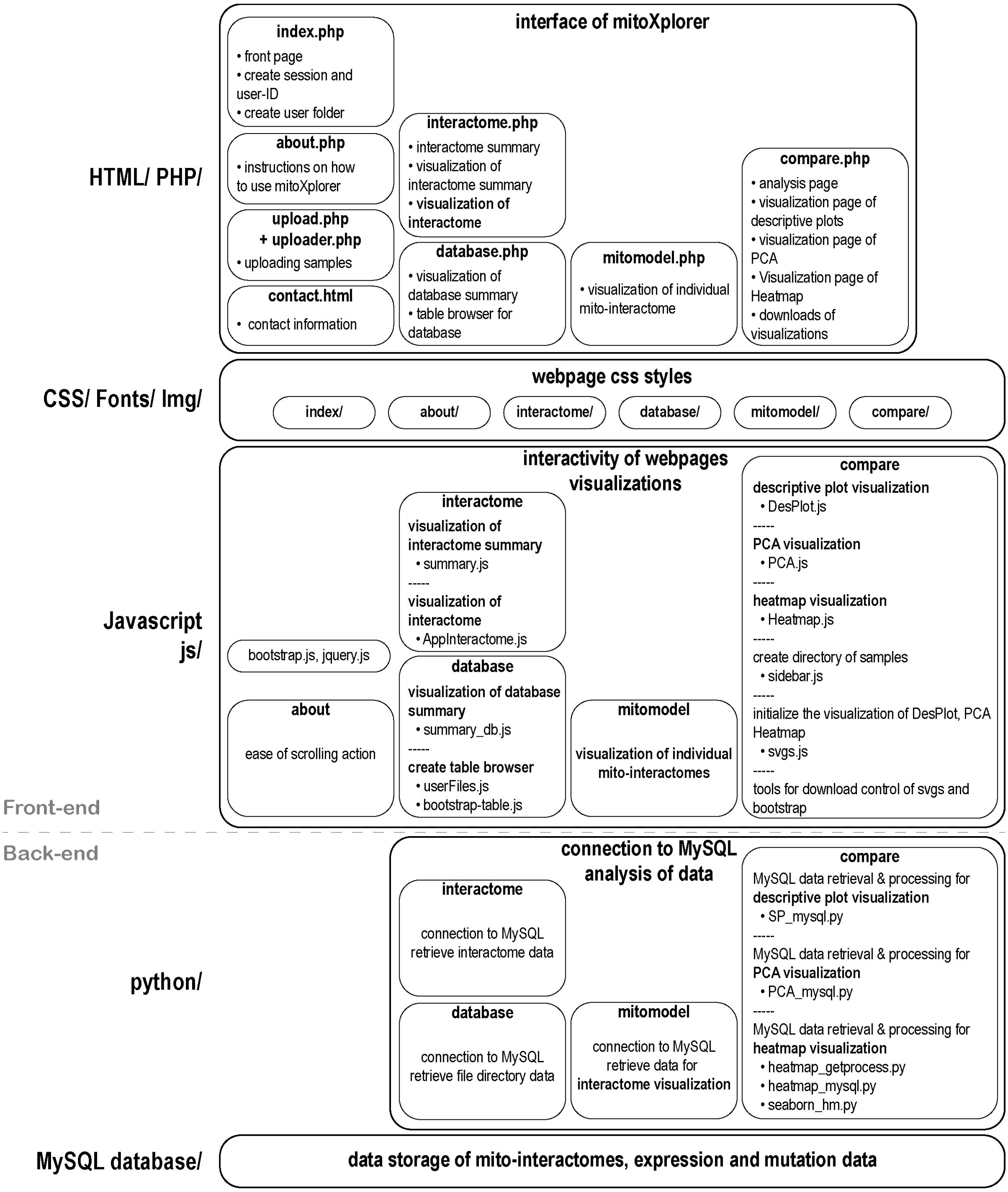

Supplementary Figure S1: programmatic skeleton of the mitoXplorer web-platform. In the *back-end*, a MySQL database stores the mito-interactomes, as well as expression and mutation data that are publicly available. User-uploaded data are stored temporarily and only available to the user. A set of python-scripts connect to the MySQL database for data retrieval of both, mito-interactomes and expression and mutation data. The mitomodel script connects to the MySQL database directly for the visualization of the Interactome View. A set of scripts perform comparative analysis, for generating Comparative Plots, Heatmap and PCA visualization. In the *front-end*, a set of javascripts handle the visualizations of the plots: the 'interactome' and 'database' scripts handle the data presentation of the

mito-interactome and the available public data for the web-site; mitomodel visualizes the Interactome View and the scripts in the compare box are responsible for visualizing Comparative Plot, Heatmap and PCA. The CSS layer handles the css-styles of the page and finally, the HTML/PHP layer creates the actual interface for the user.

Supplementary Figure S2

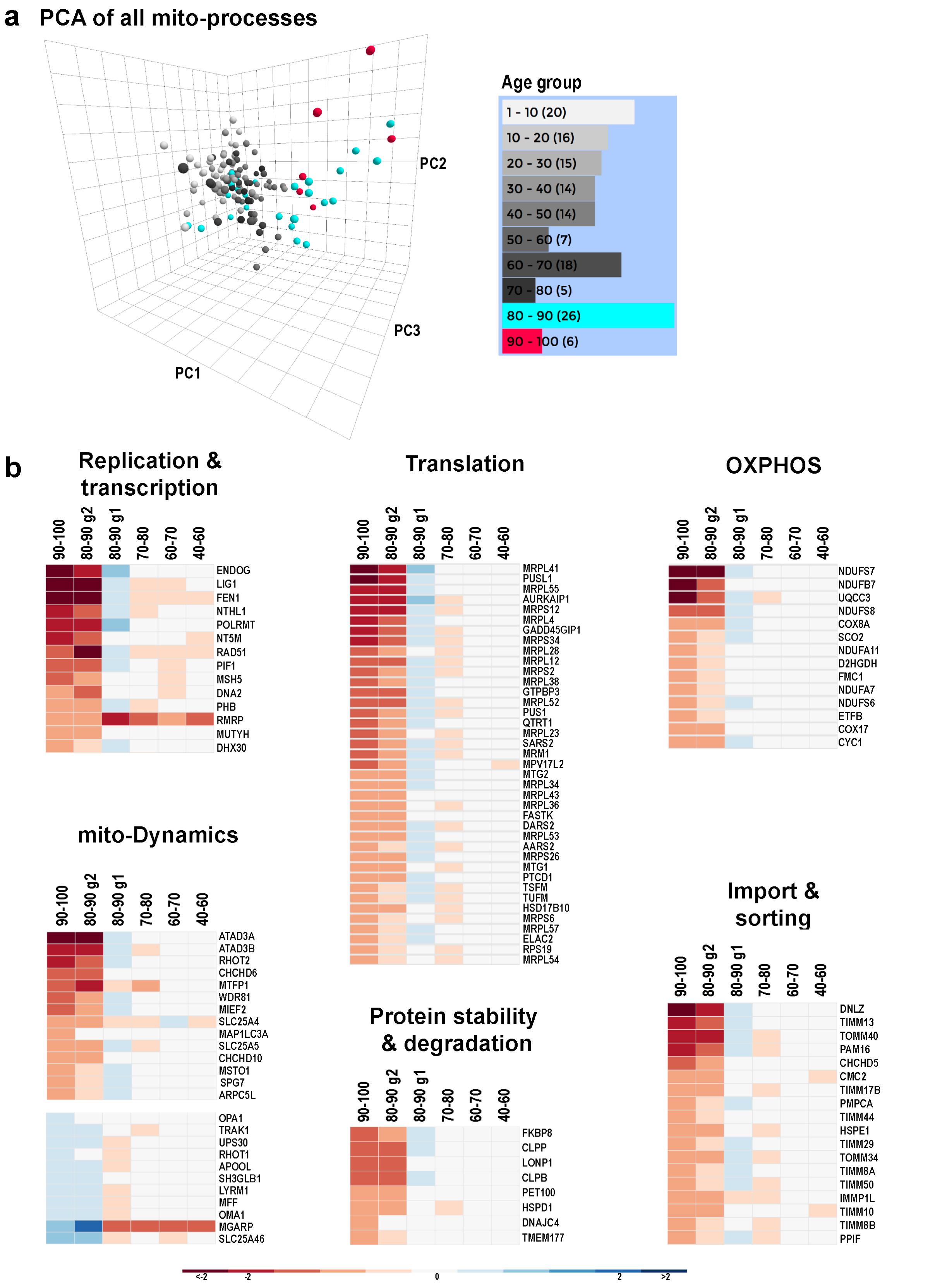

Supplementary Figure S2: Human fibroblasts from healthy donors between ages 1 and 94 reveal strong down-regulation of mito-genes in different processes at ages above 85. To demonstrate the GROUP function of mitoXplorer, we use data from human fibroblasts from different age groups (1). A number of mitochondrial processes have been implicated in ageing and ageing-related diseases,

though the precise contribution of mitochondria to ageing is so far not clear (2) . However there seems to be an increase in mtDNA mutations and resulting impaired respiratory chain functions during ageing (3). We wanted to investigate whether the expression dynamics of mito-genes changes in the ageing tissue. To this end, we uploaded data from ageing human dermal fibroblasts from a study of Fleischer et al. (1). The authors chose this tissue because of its ease of availability. Moreover, the cells in the dermal layer are not prone to environmentally-induced mtDNA mutations and thus lifestyle-induced mito-gene expression changes (4). The dataset is composed of single replicates of 133 healthy donors from age 1 to 94. We calculated log2FC from RPKM values provided by the authors and uploaded the data to mitoXplorer. **(a)** We first performed a PCA analysis of mito-gene expression over all age groups. To our surprise, the mito-gene expression profiles seemed very robust up to the age of 80. Between 80 and 90 years of age, half the age group, as well as all individuals from age group 90-100 showed a very distinct expression profiles of many mito-genes and in most mito-processes. In fact, only few mito-processes were not affected. This was the case for all individuals older than 85 years except for one male of 88 years, who grouped with younger individuals. **(b)** In order to explore the nature of the de-regulation at high age, we generated groups using the mitoXplorer GROUP function. We focused on individuals between age 40 and 100. We merged age group 40-50 and 50-60 and split age group 80-90 in one group g1 with mito-gene expression dynamics consistent with younger individuals, as well as g2 with mito-gene expression profiles similar to those found in individuals above 90 years of age. Not unexpectedly we found that a number of mito-genes were generally down-regulated.

Supplementary Figure S3

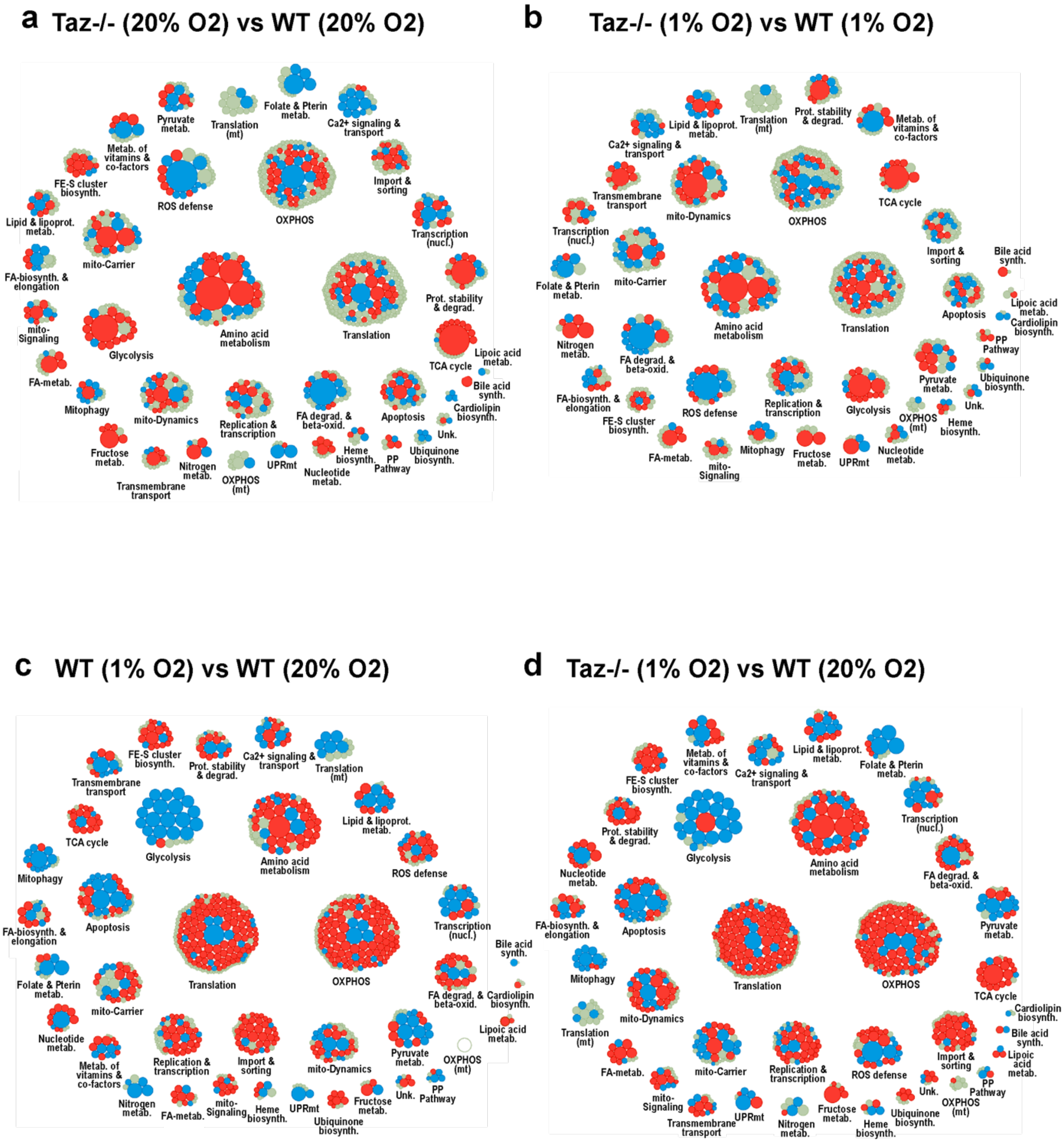

Supplementary Figure S3: Interactome Views of Tafazzin-deficient cells, as well as wild-type cells in normoxic and hypoxic conditions. (a) Tafazzin-deficient cells compared to wild-type cells. The expression dynamics from virtually all mito-processes is changed, with strongest perturbation in Amino acid metabolism, TCA cycle, Glycolysis, Fatty acid degradation & beta-oxidation, Mitochondrial carrier as well as Protein stability & degradation. **(b)** Taz-deficient cells compared to wild-type in hypoxic conditions. Changes in expression dynamics compared to normoxic conditions can for instance be observed in Mitochondrial dynamics, Transcription (nuclear), or UPRmt. **(c)** Differential expression dynamics of wild-type cells in normoxic and hypoxic conditions. In response to hypoxia, OXPHOS genes are down-regulated, while genes required for Glycolysis are induced. **(d)** Tafazzin-deficient cells in hypoxia are compared to wild-type cells in normoxic conditions. Strong changes can be observed for instance in ROS defense, Ca^{2+} Signaling & transport or UPRmt.

Supplementary Figure S4

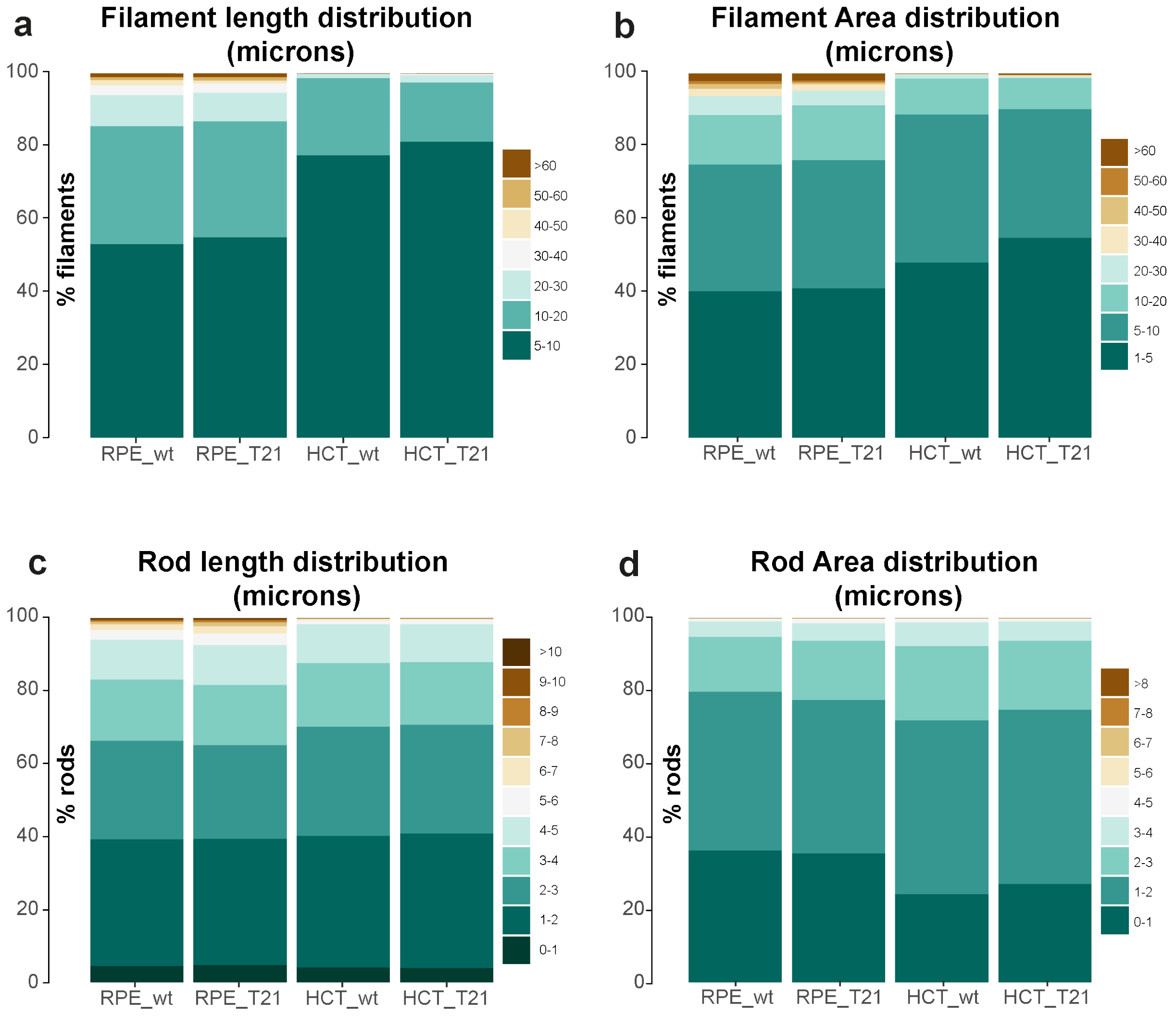

Supplementary Figure S4: Length and area distribution of filaments and rods in wild-type and T21 derived RPE1 and HCT116 cells. (a) Stacked bar-plots of filament length distribution of RPE1 wild-type (labeled RPE_wt), RPE1 21/3 (labeled RPE_T21), HCT116 wild-type (labeled HCT_wt) and HCT116 21/3 (labeled HCT_T21) cells. Overall, shorter filaments are more frequent in HCT116 than in RPE1 cells. In T21, filaments tend to be slightly shorter. **(b)** Stacked bar-plots of filament area distribution of RPE_wt, RPE_T21, HCT_wt wild-type and HCT_T21 cells. Overall, less area is occupied by filaments in HCT116 than in RPE1 cells. In HCT_T21 cells, a notably smaller area is assigned to filaments, while in RPE_T21 cells, this change is much less pronounced. **(c)** Stacked bar-plots of rod length distribution of RPE_wt, RPE_T21, HCT_wt and HCT_T21 cells. Overall, in the range between 4 and 10 microns, more rods are found in RPE1 cells. Between wild-type and T21 cells, no real length difference is observable. **(d)** Stacked bar-plots of rod area distribution of RPE_wt, RPE_T21, HCT_wt and HCT_T21 cells. Overall, there is a tendency of slightly larger rod areas in HCT116 cells. In HCT116 cells, rods seem to occupy slightly smaller areas when carrying the extra copy of chromosome 21. Data were averaged over the two clones of RPE_T21 and HCT_T21, respectively.

Supplementary Figure S5

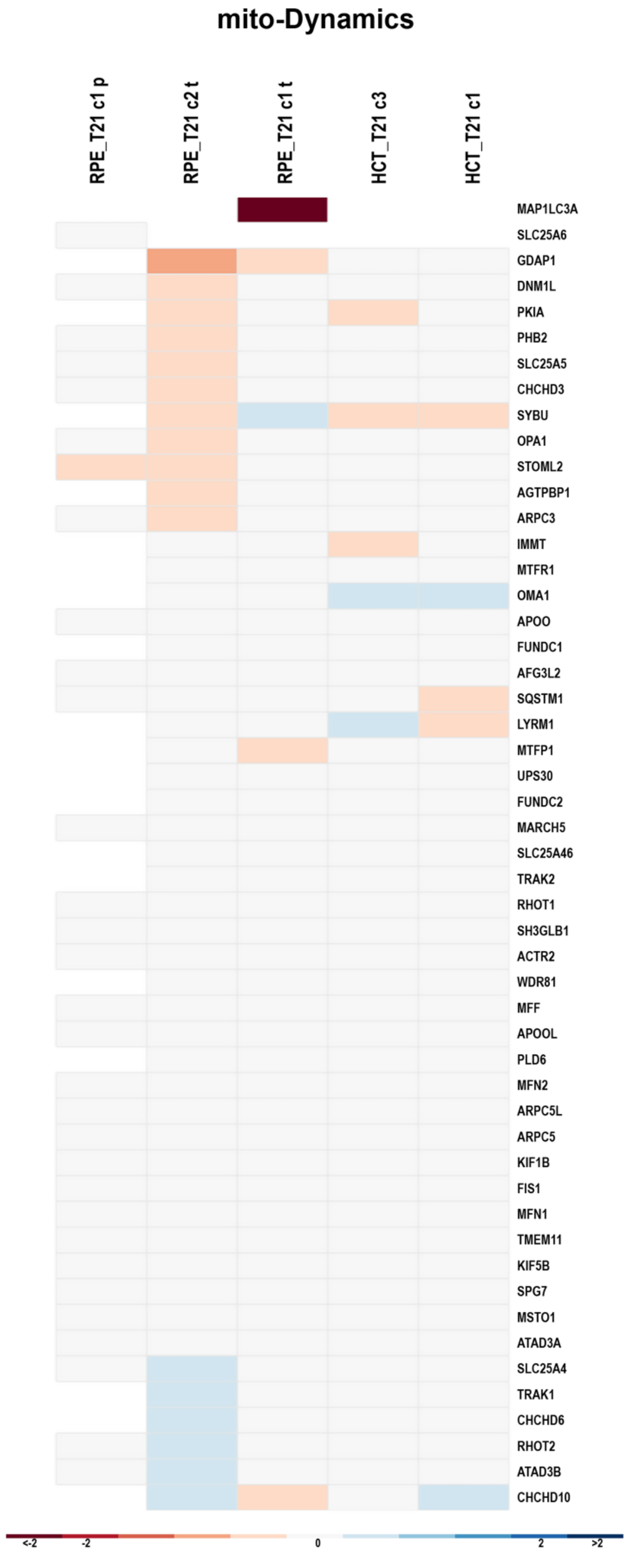

Supplementary Figure S5: The mito-gene GDAP1 is consistently down-regulated in RPE_T21 clones. Shown is the heatmap of the Comparative Analysis page of mitoXplorer. Both clones of the RPE21_T21 cells show significant down-regulation of the GDAP1 gene; clones 1 and 3 of HCT_T21 cells also show slight, though statistically non-significant reduction of this gene (RPE_T21 c1: log2FC = -1.103; RPE_T21 c2: log2FC = -1.604; HCT_T21 c1: -0.238; HCT_T21 c3: -0.059).

Supplementary Figure S6

a Translation

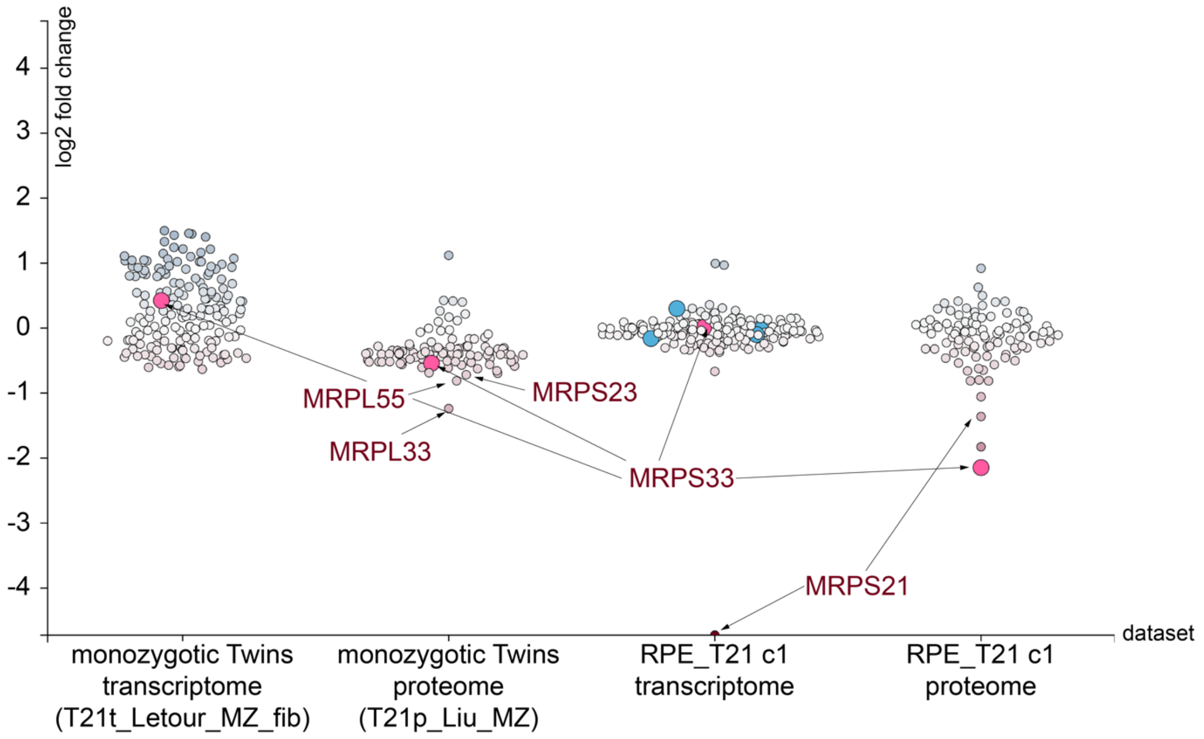

b Oxidative phosphorylation

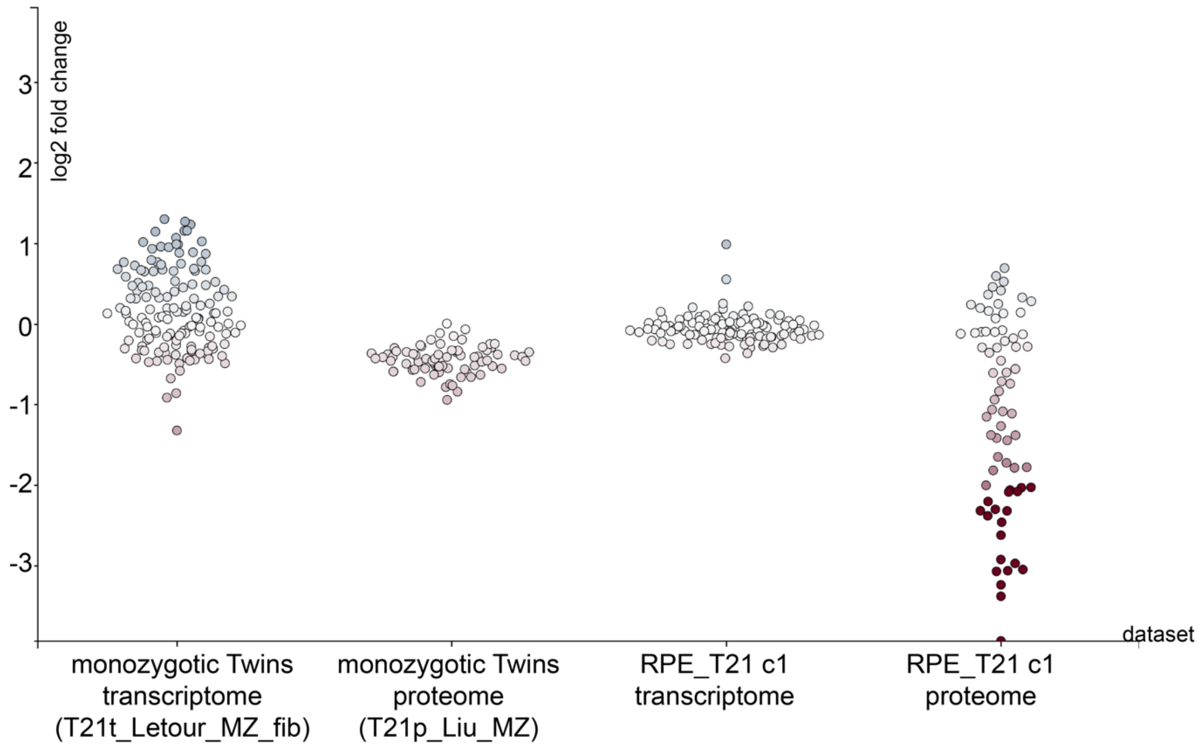

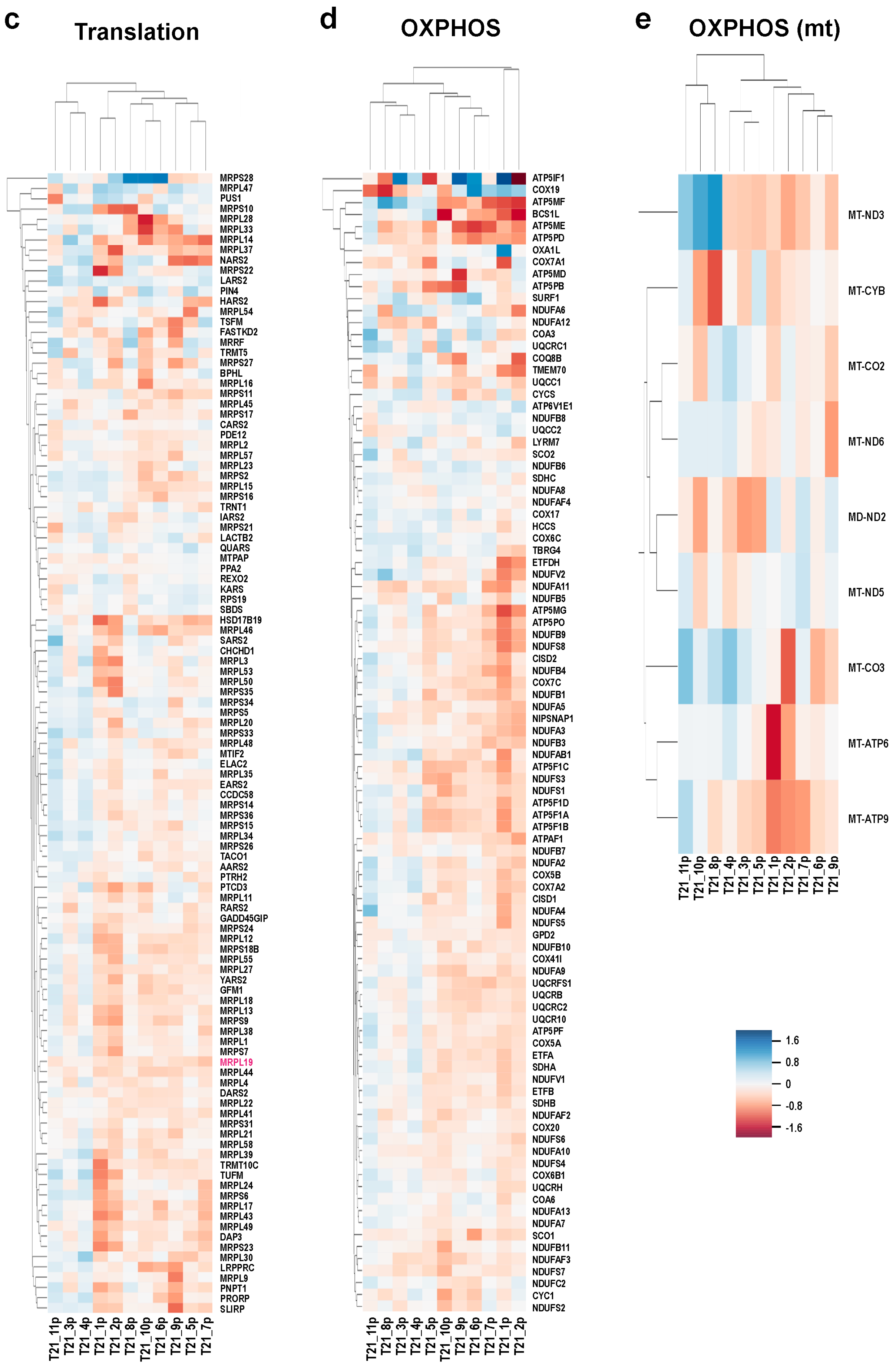
c
Translation
MRPS28
MRPL47
PUS1
MRPS10
MRPL28
MRPL33
MRPL14
MRPL37
NARS2
MRPS22
LARS2
PIN4
HARS2
MRPL54
TSFM
FASTKD2
MRRF
TRMT5
MRPS27
BPHL
MRPL16
MRPS11
MRPL45
MRPS17
CARS2
PDE12
MRPL2
MRPL57
MRPL23
MRPS2
MRPL15
MRPS16
TRNT1
IARS2
MRPS21
LACTB2
QUARS
MTPAP
PPA2
REXO2
KARS
RPS19
SBDS
HSD17B19
MRPL46
SARS2
CHCHD1
MRPL3
MRPL53
MRPL50
MRPS35
MRPS34
MRPS5
MRPL20
MRPS33
MRPL48
MTIF2
ELAC2
MRPL35
EARS2
CCDC58
MRPS14
MRPS36
MRPS15
MRPL34
MRPS26
TACO1
AARS2
PTRH2
PTCD3
MRPL11
RARS2
GADD45GIP
MRPS24
MRPL12
MRPS18B
MRPL55
MRPL27
YARS2
GFM1
MRPL18
MRPL13
MRPS9
MRPL38
MRPL1
MRPS7
MRPL19
MRPL44
MRPL4
DARS2
MRPL22
MRPL41
MRPS31
MRPL21
MRPL58
MRPL39
TRMT10C
TUFM
MRPL24
MRPS6
MRPL17
MRPL43
MRPL49
DAP3
MRPS23
MRPL30
LRPPRC
MRPL9
PNPT1
PRORP
SLIRP
T21_11p
T21_3p
T21_4p
T21_1p
T21_2p
T21_8p
T21_10p
T21_6p
T21_9p
T21_5p
T21_7p
d
OXPHOS
ATP5IF1
COX19
ATP5MF
BCS1L
ATP5ME
ATP5PD
OXA1L
COX7A1
ATP5MD
ATP5PB
SURF1
NDUFA6
NDUFA12
COA3
UQCRC1
COQ8B
TMEM70
UQCC1
CYCS
ATP6V1E1
NDUFB8
UQCC2
LYRM7
SCO2
NDUFB6
SDHC
NDUFA8
NDUFAF4
COX17
HCCS
COX6C
TBRG4
ETFDH
NDUFV2
NDUFA11
NDUFB5
ATP5MG
ATP5PO
NDUFB9
NDUFS8
CISD2
NDUFB4
COX7C
NDUFB1
NDUFA5
NIPSNAP1
NDUFA3
NDUFB3
NDUFAB1
ATP5F1C
NDUFS3
NDUFS1
ATP5F1D
ATP5F1A
ATP5F1B
ATPAF1
NDUFB7
NDUFA2
COX5B
COX7A2
CISD1
NDUFA4
NDUFS5
GPD2
NDUFB10
COX4I1
NDUFA9
UQCRFS1
UQCRB
UQCRC2
UQCR10
ATP5PF
COX5A
ETFA
SDHA
NDUFV1
ETFB
SDHB
NDUFAF2
COX20
NDUFS6
NDUFA10
NDUFS4
COX6B1
UQCRH
COA6
NDUFA13
NDUFA7
SCO1
NDUFB11
NDUFAF3
NDUFS7
NDUFC2
CYC1
NDUFS2
T21_11p
T21_8p
T21_3p
T21_4p
T21_5p
T21_10p
T21_9p
T21_6p
T21_7p
T21_1p
T21_2p
e
OXPHOS (mt)
MT-ND3
MT-CYB
MT-CO2
MT-ND6
MD-ND2
MT-ND5
MT-CO3
MT-ATP6
MT-ATP9
T21_11p
T21_10p
T21_8p
T21_4p
T21_3p
T21_5p
T21_1p
T21_2p
T21_7p
T21_6p
T21_9p
1.6
0.8
0
-0.8
-1.6

Supplementary Figure S6: mitoXplorer analysis of the mito-processes ‘Translation’, ‘Oxidative Phosphorylation’ and ‘Oxidative Phosphorylation (mt)’ of monozygotic twins discordant for T21 and 11 unrelated trisomy 21 patients. (a) The mRNA of mitoribosome small subunit component MRPS21 is strongly down-regulated in RPE_T21 cells. In monozygotic twins discordant for T21, other subunits of the small and large mitoribosome are down-regulated (T21_MZ fibroblasts: T21_Letour_MZ_fib, T21_Liu_MZ). Mitoribosome proteins are more mildly affected in T21_MZ fibroblasts. **(b)** Oxidative phosphorylation components encoded in the nucleus are downregulated on protein level in both, RPE_T21, as well as T21_MZ fibroblasts, whereby deregulation is milder in T21_MZ. In both conditions, the Oxidative phosphorylation transcriptome is mostly unaffected. **(c - e)** Mito-protein expression dynamics from fibroblasts of unrelated trisomy 21 patients in the mito-processes ‘Translation’ **(c)**, ‘Oxidative phosphorylation’ **(d)** and ‘Oxidative phosphorylation (mt)’ **(e)**. Protein levels of mitochondrial- and nuclear-encoded subunits of the respiratory chain is very heterogeneous between unrelated individuals with trisomy 21; yet, in most trisomy 21 patients several mitochondrial-, as well as nuclear-encoded subunits of the respiratory chain are down-regulated on protein level. A similar heterogeneity can be observed for protein expression levels involved in translation of mitochondrial proteins **(c)**. At least a few proteins involved in ‘Translation’ are down-regulated in each individual with trisomy 21. The only consistently, though sometimes only mildly reduced protein in this mito-process is mitoribosomal protein MRPL19 (highlighted in red), while MRPS21 shows variable expression patterns in unrelated trisomy 21 patients. Protein expression data are taken from Liu et al. (5). The log2FC of individual trisomy 21 patients versus the average of 11 unrelated healthy individuals was calculated using the log2 of the SWATH-MS intensities provided by the authors.

Supplementary Figure S7

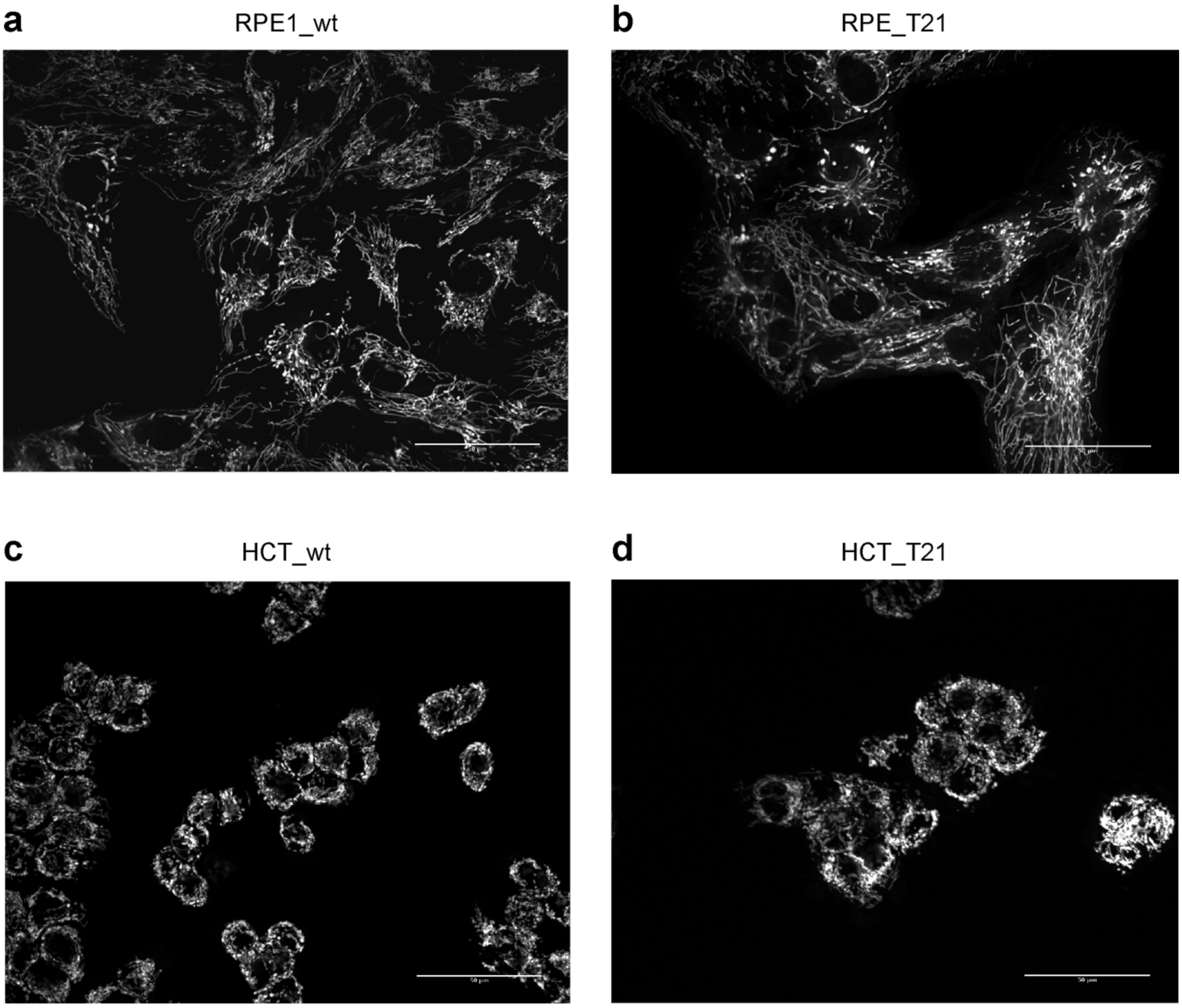

Supplementary Figure S7: Mitochondrial network of wild-type and T21 cells. MitoTracker stainings **(a-d)** of RPE_wt **(a)** and RPE_T21 **(b)**, as well as HCT_wt **(c)** and HCT_T21 **(d)**. **(a, b)** The mitochondrial network is largely intact in RPE_T21 cells, with only slightly lower percentage filaments and an increased number of swollen mitochondria. **(c, d)** In HCT116 cells, the mitochondrial network is overall less abundant, with more rod-like and fragmented mitochondria (puncta). With trisomy 21, cells show an even more pronounced presence of rods at the cost of longer filaments, as well as more puncta and swollen mitochondria. The scale bar is 50 µm. Mitochondria were stained with MitoTracker deep Red FM from Invitrogen. Staining was done in 96-well plates. The cells were incubated for 30 min at 30°C with 100 nM MitoTracker dye prior to fixation. Cells were fixed with 3% PFA in DMEM for 5 min at room temperature. After washing with 1xPBS, 1xPBS with 0.01% sodium azide was added. Plates were stored at 4°C in the dark. Imaging was carried out on an inverted Zeiss Observer.Z1 microscope with a spinning disc and 473 nm, 561 nm and 660 nm argon laser lines. The images were captured automatically on multiple focal planes (step size 700 nm) with a 40x magnification air objective. Image stacks were Z-projected using Fiji for further analysis.

References

1. Fleischer,J.G., Schulte,R., Tsai,H.H., Tyagi,S., Ibarra,A., Shokhirev,M.N., Huang,L., Hetzer,M.W. and Navlakha,S. (2018) Predicting age from the transcriptome of human dermal fibroblasts. *Genome Biol.*, **19**, 221–8.

2. Bratic,I. and Trifunovic,A. (2010) Mitochondrial energy metabolism and ageing. *Biochim. Biophys. Acta*, **1797**, 961–967.

3. Park,C.B. and Larsson,N.-G. (2011) Mitochondrial DNA mutations in disease and aging. *J. Cell Biol.*, **193**, 809–818.

4. Stout,R. and Birch-Machin,M. (2019) Mitochondria's Role in Skin Ageing. *Biology (Basel)*, **8**, 29.

5. Liu,Y., Borel,C., Li,L., Müller,T., Williams,E.G., Germain,P.-L., Buljan,M., Sajic,T., Boersema,P.J., Shao,W., *et al.* (2017) Systematic proteome and proteostasis profiling in human Trisomy 21 fibroblast cells. *Nat Commun*, **8**, 1212.
